# Next-generation MRI scanner designed for ultra-high-resolution human brain imaging at 7 Tesla

**DOI:** 10.1038/s41592-023-02068-7

**Published:** 2023-11-27

**Authors:** David A. Feinberg, Alexander J. S. Beckett, An T. Vu, Jason Stockmann, Laurentius Huber, Samantha Ma, Sinyeob Ahn, Kawin Setsompop, Xiaozhi Cao, Suhyung Park, Chunlei Liu, Lawrence L. Wald, Jonathan R. Polimeni, Azma Mareyam, Bernhard Gruber, Rüdiger Stirnberg, Congyu Liao, Essa Yacoub, Mathias Davids, Paul Bell, Elmar Rummert, Michael Koehler, Andreas Potthast, Ignacio Gonzalez-Insua, Stefan Stocker, Shajan Gunamony, Peter Dietz

**Affiliations:** 1 Erwin Hahn 7T MRI Laboratory, Henry H. Wheeler Brain Imaging Center, Helen Wills Neuroscience Institute, University of California, Berkeley, Berkeley, CA, USA.; 2 Advanced MRI Technologies, Sebastopol, CA, USA.; 3 Radiology and Biomedical Imaging, University of California, San Francisco, San Francisco, CA, USA.; 4 San Francisco Veteran Affairs Health Care System, San Francisco, CA, USA.; 5 A. A. Martinos Center for Biomedical Imaging, Department of Radiology, Massachusetts General Hospital, Charlestown, MA, USA.; 6 Harvard-MIT Health Sciences and Technology, MIT, Cambridge, MA, USA.; 7 Faculty of Psychology and Neuroscience, Maastricht University, Maastricht, the Netherlands.; 8 Siemens Medical Solutions, Malvern, PA, USA.; 9 Radiological Sciences Laboratory, Stanford University, Stanford, CA, USA.; 10 Department of Computer Engineering, Chonnam National University, Gwangju, Republic of Korea.; 11 Department of ICT Convergence System Engineering, Chonnam National University, Gwangju, Republic of Korea.; 12 BARNLabs, Muenzkirchen, Austria.; 13 German Center for Neurodegenerative Diseases (DZNE), Bonn, Germany.; 14 Center for Magnetic Resonance Research, University of Minnesota, Minneapolis, MN, USA.; 15 Computer Assisted Clinical Medicine, Medical Faculty Mannheim, Heidelberg University, Heidelberg, Germany.; 16 Siemens Healthcare GmbH, Erlangen, Germany.; 17 Imaging Centre of Excellence, University of Glasgow, Glasgow, UK.; 18 MR CoilTech Limited, Glasgow, UK.

## Abstract

To increase granularity in human neuroimaging science, we designed and built a next-generation 7 Tesla magnetic resonance imaging scanner to reach ultra-high resolution by implementing several advances in hardware. To improve spatial encoding and increase the image signal-to-noise ratio, we developed a head-only asymmetric gradient coil (200 mT m^−1^, 900 T m^−1^s^−1^) with an additional third layer of windings. We integrated a 128-channel receiver system with 64- and 96-channel receiver coil arrays to boost signal in the cerebral cortex while reducing g-factor noise to enable higher accelerations. A 16-channel transmit system reduced power deposition and improved image uniformity. The scanner routinely performs functional imaging studies at 0.35–0.45 mm isotropic spatial resolution to reveal cortical layer functional activity, achieves high angular resolution in diffusion imaging and reduces acquisition time for both functional and structural imaging.

Functional magnetic resonance imaging (fMRI) has become the mainstay of human imaging in neuroscience. Typical fMRI studies use an isotropic spatial resolution on the order of 3.0 mm (27 μl voxel volume) to inform many questions about human brain functional organization. However, much higher resolution would enable exploration of neural circuits at the scale of cerebral columns and cortical layers. Human fMRI at about 0.8 mm isotropic resolution (0.5 μl)^1-3^ has been used to study columnar^4-9^ and laminar organization^10-16^ (mesoscale circuitry), which has been achieved by limiting the image field of view (FOV) to small targeted regions of cerebral cortex. With human cortex thickness varying between 1.5 and 4.5 mm (refs. 17,18) and with cortical columnar features being 0.6–1.0 mm, higher resolution is needed to adequately sample cortical layers and columns and to minimize partial volume averaging of surrounding white matter and cerebrospinal fluid (CSF). Advancing the resolution to the next level, for example to 0.3–0.6 mm (0.03–0.2 μl)^1^, would allow the six neuronal layers of the human cerebral cortex to be adequately sampled. Reaching mesoscale spatial resolution is also beneficial in structural and diffusion imaging, which can reveal depth-dependent organization of axonal fiber tracks^19-21^ when performed at higher spatial and angular resolutions.

Toward the goal of reaching higher spatial resolution MRI for neuroscience, we designed and developed a next-generation (NexGen) 7 Tesla (7 T) human brain scanner to perform a wide range of MRI image acquisition techniques used in neuroscience, including functional, diffusion, physiological and structural imaging techniques. Several high-performance system components were built and adapted at the ultra-high field of 7 T, which was critical to achieve higher resolution in brain imaging.

Over the years, improvements to major MRI scanner system components (for example, magnetic field gradient coils^22-24^ and radiofrequency (RF) receiver array coils^25^) have led to higher signal-to-noise ratio (SNR) and higher resolution imaging or faster image acquisition (acceleration). However, such hardware improvements were typically developed in isolation from one another and at less challenging, lower field strengths (for example, 3 T). To achieve higher resolution, we took advantage of the higher signal afforded by 7 T ultra-high field scanners in which we integrated hardware systems including a head-only magnetic gradient coil, a receiver system, acquisition computer, receiver coils and transmit coils. Critically, these system components were designed, built and optimized concurrently. Our approach of comprehensive system design and integration overcame several physical challenges including space constraints, system cooling, peripheral nerve stimulation (PNS), eddy currents and the interaction of higher forces and torques that are produced by strong magnetic gradients at ultra-high magnetic field. The improvements of the gradient coil combined with the larger channel count receiver arrays enable higher acceleration of data capture and higher SNR. In aggregate, the higher signal^26^ and greater blood oxygenation level-dependent (BOLD) contrast of 7 T coupled with higher resolution, SNR and faster signal acquisition of the NexGen 7 T scanner enable improved diffusion imaging and mesoscale imaging in vivo.

## Results

### Head gradient coil

**Fig. 1 ∣:**
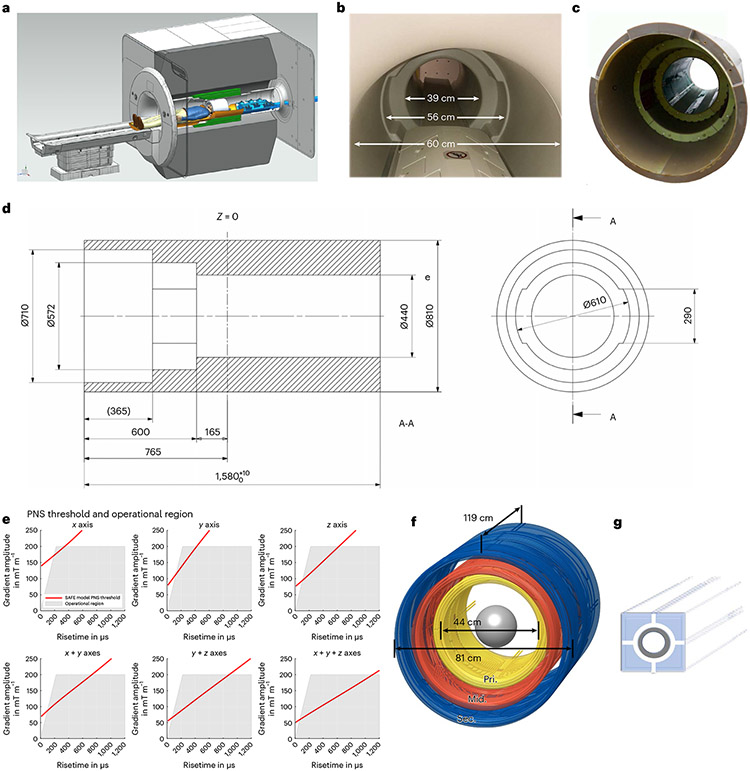
NexGen 7 T scanner. **a**, Cross-sectional rendering of the scanner showing the Impulse gradient coil (green), the receiver–transmit coil connectors attached to the coil interface box with energy chain extending out of the magnet (blue) and a receiver–transmit coil (white) resting on extension of the movable bed (brown). **b**, Photo of the scanner with the acoustic bore liner giving a 39 cm diameter head region, 56 cm wide shoulder spaces and 60 cm diameter body bore. **c**, Photograph of the Impulse gradient coil. **d**, Cross-sectional dimensions of the Impulse gradient coil showing key dimensions (mm). The three coil axes are combined in each of the three layers of windings (primary (Pri.), middle (Mid.), secondary (Sec.)) and the shoulder cutouts are in the *y* axis middle layer. **e**, PNS threshold limits in scanner operational region determined by maximum gradient amplitude and rise time (SR) below PNS thresholds. The red line shows the SAFE model threshold^31^ used during normal scanner operation. **f**, A 3D layout of the gradient coil showing the three layers of coil winding (primary, middle, secondary). **g**, Diagrammatic rendering of a segment of the (gray) stainless-steel cooling tubes integrated into conductive windings surrounded by copper filament conductors.

Standard shielded gradient coils include two layers of conductive wiring: the primary inner layer creates linear magnetic fields inside the coil for spatial encoding images while the secondary outer winding layer cancels the external magnetic field to reduce eddy currents in the surrounding superconducting magnet. Our high-performance ‘Impulse’ head-only asymmetric gradient coil incorporates an intermediate third layer of wire winding (Fig. 1) providing additional degrees of freedom needed for simultaneous reduction of PNS^27^ and optimization of gradient field quality, mechanical resonances and torque.

The Impulse gradient coil achieved very fast switching of the gradients (slew rate, SR) of 900 T m^−1^ s^−1^ and maximum amplitude of gradient (Gmax) of 200 mT m^−1^ (Table 1). By contrast, the standard 7 T whole-body gradient coils can achieve an SR of 200 T m^−1^ s^−1^ and Gmax of 80 mT m^−1^. The performance and peak power achievable by the Impulse gradient system, as defined by the product of SR and Gmax^24^, is an order of magnitude greater than the current standard 7 T systems and about five times the performance of the existing head-only gradient coil operating at 7 T, ‘AC84’ (80 mT m^−1^, 400 T m^−1^ s^−1^). Physiological modeling^28^ used to optimize the wiring pattern of the three-layer gradient coil^29^ (Fig. 1) minimized PNS. Thus, the Impulse gradient coil can be used at higher SR, and is less hampered by PNS limitations in gradient-demanding sequences, particularly in echo-planar imaging (EPI). By contrast, previous 7 T head-only gradient coils use two winding layers and are limited by PNS to an SR of 400–500 T m^−1^ s^−1^ (ref. 30), while standard body gradient coils are limited by cardiac stimulation to roughly 200 T m^−1^ s^−1^. The Impulse gradient operating curves are limited by PNS, using an adapted model^31^ based on healthy participants’ stimulation thresholds described in the ‘PNS supervision’ section in the Methods (Fig. 1e). The design of the gradient coil cooling system also differs from conventional gradient coils that use hollow copper conductor filaments, whereas the Impulse coil’s cooling system is designed with stainless-steel tubing surrounded by conductive filaments (Fig. 1g).

**Extended Data Fig. 1 ∣:**
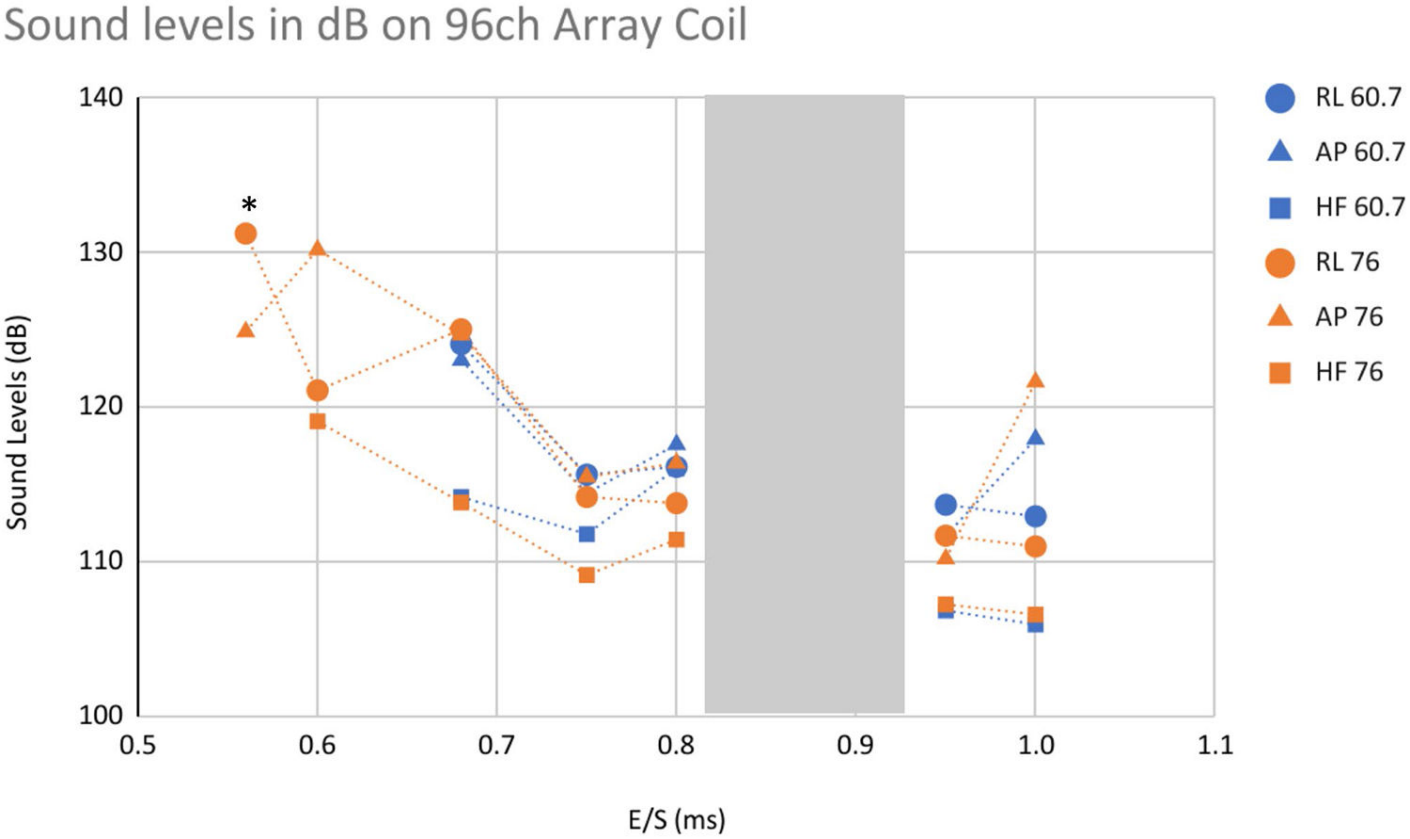
Sound pressure levels. Measured using a high-resolution EPI sequence using two gradient strengths, typically used for neuroimaging studies, at a range of echo spacings using three orthogonal readout directions, and showed sound levels within prescribed safety limits (< 99 dB) when using 33 dB attenuation earplugs. (*)Approximate dB measures at 0.55 ms echo spacing (E/S) due to sound meter clipping and HF 76 did not run at 0.55 ms E/S due to stimulation warning.

The sound pressure dependent acoustic noise of the Impulse gradient coil was lower than that of the standard whole-body SC72 gradient coil when operated at similar maximum gradient strength. Sound levels reached a value of 120.6 dB(A) with all axes combined (Table 1), which is within the safety limits when using at least 28 dB attenuation earplugs. Sound levels measured using a high-resolution EPI sequence at a range of echo spacings (ESs) on three orthogonal readout directions also showed sound levels within prescribed safety limits (less than 99 dB) when using 33 dB attenuation earplugs (Extended Data Fig. 1).

### Receiver system

The scanner supports up to 128 receiver channels to allow smaller coil loop diameters in a receiver coil array covering the entire head. We found that reducing the diameter of RF coil loops to about 4 cm at 7 T resulted in a higher signal in the human neocortex^32^.

**Extended Data Fig. 2 ∣:**
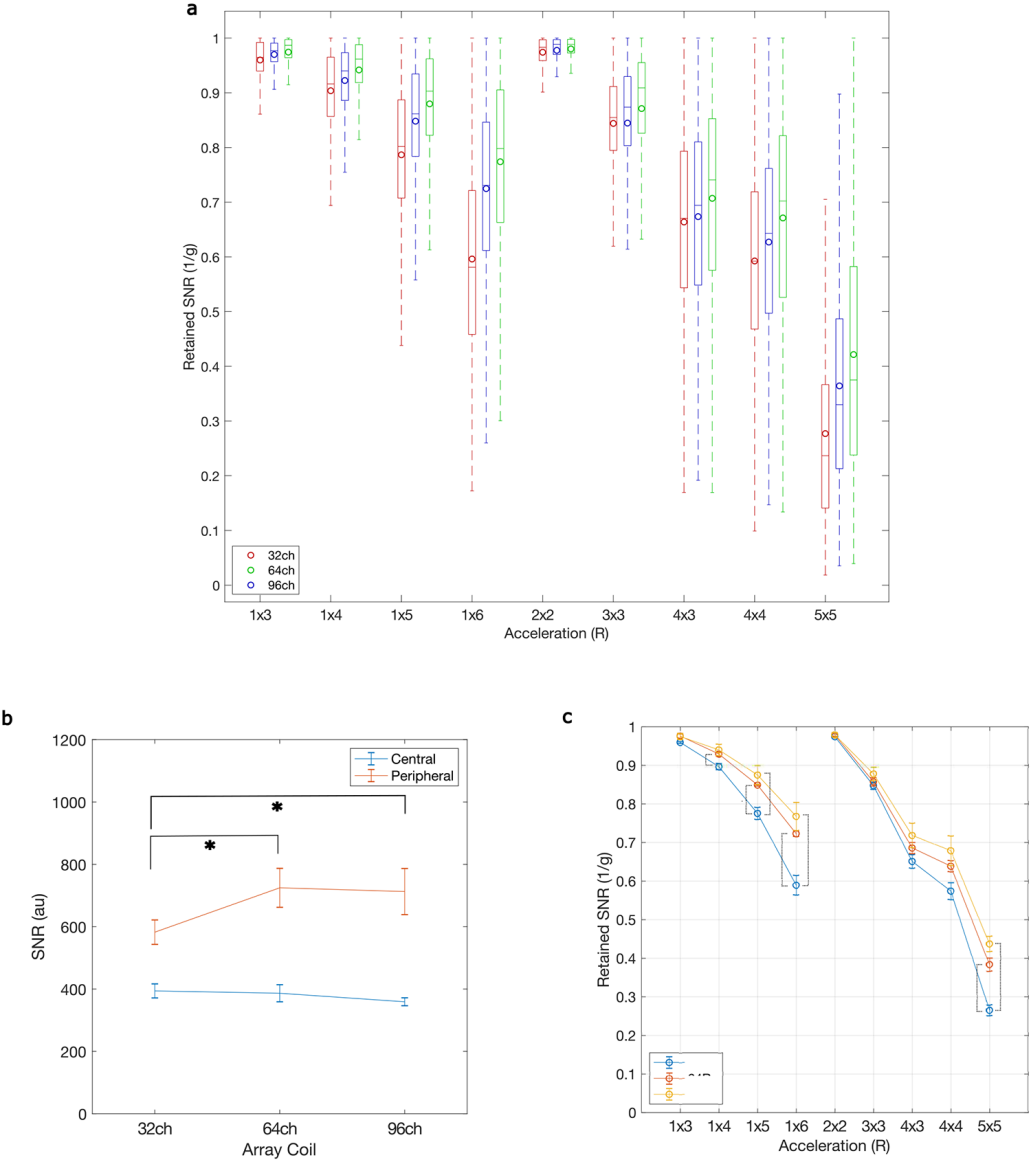
RF Array Coil Performance. A) Histogram of retained SNR (1/G) for different accelerations for 3 arrays coils (32 ch, 64ch, 96ch). Boxes show median, 25th and 75th percentile values (n = 219233 voxels across a whole brain ROI). Circles show mean values. Whiskers show 1.5 times the interquartile range. B) Across 3 subjects, the 64Rx (paired sample t-test, p = .0144) and 96Rx (paired sample t-test, p = .0272) showed significantly higher SNR in the periphery of the brain when compared to the 32Rx. The central ROI did now show higher SNR for higher channel count arrays. Data are presented as mean values +/− standard deviation (SD). C) Across 3 subjects, the 64Rx showed higher retained SNR (1/g) than the 32Rx at accelerations of 1×4, 1×5, 1×6 and 5×5 (paired sample t-tests, p = .0433,.0114,.0085,.0067, Bonferroni corrected). The 96Rx showed higher retained SNR (1/g) than the 32Rx at accelerations of 1×5, 1×6 and 5×5 (paired sample t-test, p = .0371,.0202,.0022, Bonferroni corrected). Data are presented as mean values +/− SD.

**Fig. 2 ∣:**
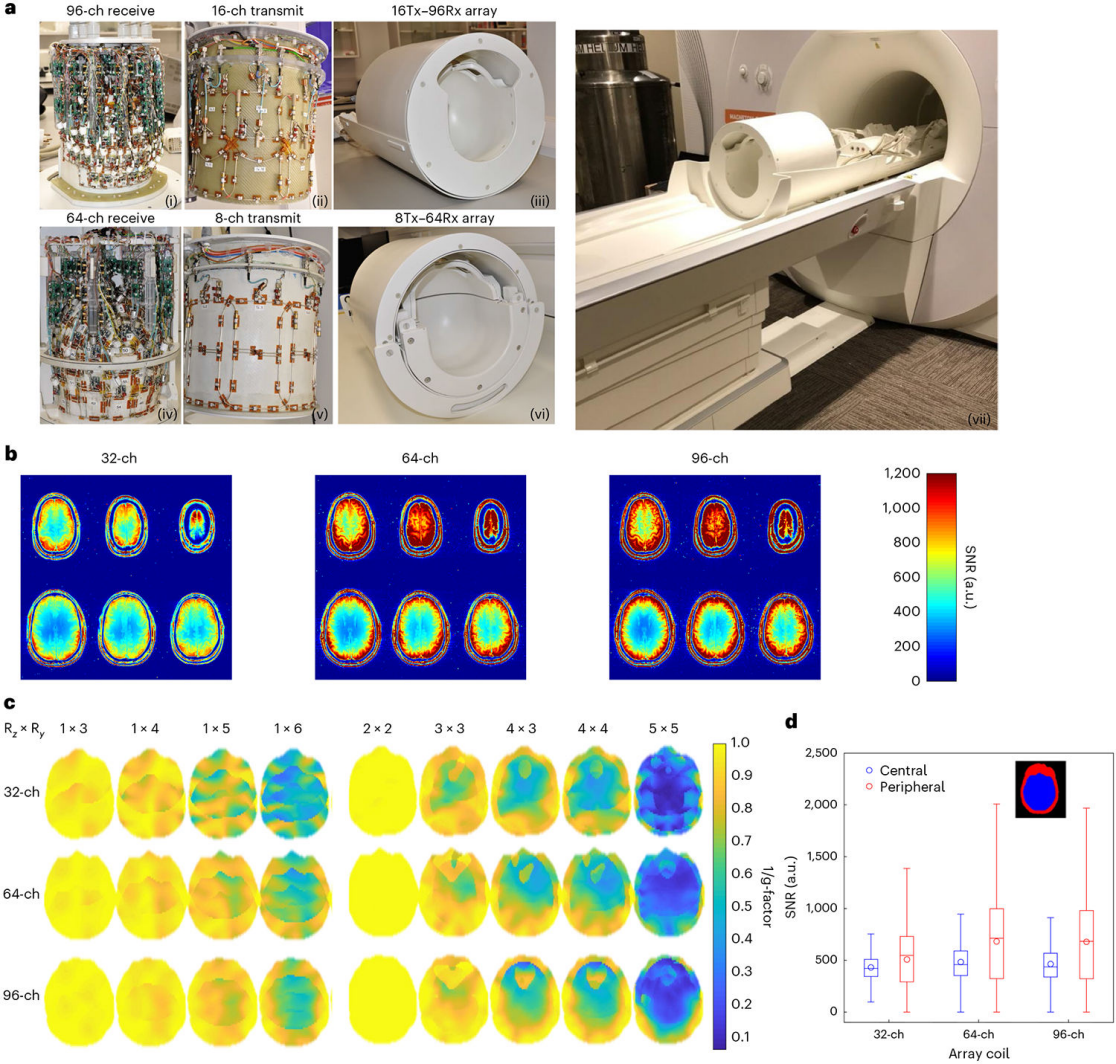
High-density receiver array coils and transmit array coils. **a**, The top row shows a 16-ch Tx and 96-ch Rx coil: a photograph of the completed 96-ch array (i), the 16-ch dual-row transmit array (ii) and the fully assembled 16-ch Tx, 96-ch Rx coil (iii). The bottom row shows an 8-ch Tx, 64-ch Rx coil: a photograph of the 64-ch receive array (iv), the 8-ch transmit array (v) and the fully assembled 8-ch Tx, 64-ch Rx coil (vi). The scanner’s movable table is specially designed to incorporate the RF array coils (vii). **b**, Comparison of receiver coil SNR maps between standard 32-ch coil and the 64- and 96-ch coil measured in the same participant. **c**, Retained SNR (1/g maps) for a range of accelerations (R) on the 64-ch Rx and 96-ch Rx array compared to a standard 32-ch array. **d**, Boxplots of SNR distributions in a central and peripheral ROI (*n* = 363,171 and 422,316 voxels, respectively) for each array coil (right panel). Boxes show the median, 25th and 75th percentile values. Circles show mean values. Whiskers show 1.5 times the interquartile range.

Two RF receiver (Rx)–transmit (Tx) coil arrays are currently operating on our system: a 64-channel receive array coil (64-ch Rx, 8-ch Tx) and a 96-channel coil (a 96-ch Rx-16-ch Tx) (Fig. 2). Both were used to achieve higher signal in the cortex compared to the standard 32-ch Rx coil available on conventional 7 T scanners. In comparison to the 32-ch Rx coil, our results show roughly 30% improvement in SNR in cortical regions (Fig. 2b,d). Across three participants, the 64-ch Rx and 96-ch Rx showed significantly higher SNR in the periphery of the brain when compared to the 32-ch Rx (paired sample *t*-tests, *P* = 0.014 and *P* = 0.027, respectively; Extended Data Fig. 2b). The center of the coil array did not show higher SNR for higher channel count arrays. Our larger receiver arrays with reduced coil loop size support higher acceleration factors by reducing the g-factor SNR noise penalty for the 96-ch and 64-ch Rx coils compared to the 32-ch Rx array coil (Fig. 2c and Extended Data Fig. 2a,c).

### Gradient coil performance and resolution comparisons

**Fig. 3 ∣:**
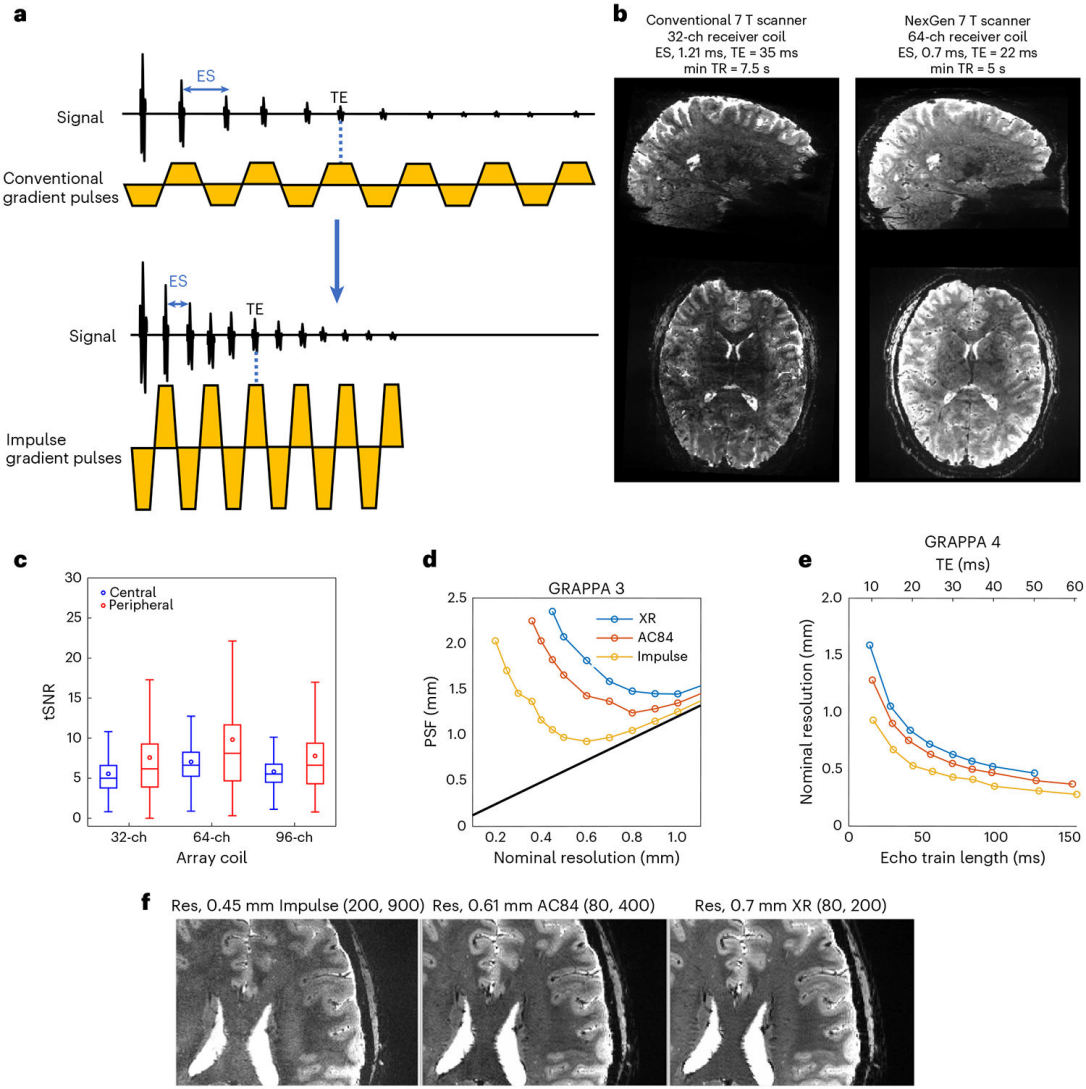
EPI on the NexGen 7 T. **a**, EPI pulse sequence diagram shows readout gradient pulses with conventional versus higher amplitude and faster SR that reduces ES. **b**, Comparison of EPI image quality at 0.6 mm isotropic resolution covering the brain on the conventional 7 T scanner (MAGNETOM 7 T Plus) and the NexGen 7 T scanner with the Impulse head gradient coil using the same acquisition parameters: GRAPPA × SMS = 4 × 3, partial Fourier 6/8, 216 slices, matrix size 320 × 320. The left shows the conventional 7 T (80 mT m^−1^, 200 T m^−1^ s^−1^, 32-ch Rx, 8-ch Tx). The right shows the NexGen 7 T (200 mT m^−1^, 900 T m^−1^ s^−1^, 64-ch Rx, 8-ch Tx coil). **c**, Box plot of temporal SNR within a central and peripheral ROI (*n* = 3,333,301 and 3,980,755 voxels, respectively). Boxes show median, 25th and 75th percentile values. Circles show mean values. Whiskers show 1.5 times the interquartile range. **d**, PSF on EPI image phase-encoded axis due to T2* decay for a given resolution achievable at three different gradient coil performances using GRAPPA acceleration of 3. **e**, Achievable nominal resolution at a given TE with same echo train duration with the same T2* signal decay for three different gradient coil performances using GRAPPA acceleration of 4 and 6/8 partial Fourier. **f**, EPI images, at maximum achievable resolution (Res) at TE 26 ms for three different gradient coils. The highest achievable isotropic volumetric resolutions are 0.09 μl (0.45 mm isotropic voxel), 0.23 μl (0.61 mm isotropic) and 0.343 μl (0.7 mm isotropic). Differences in Gmax and SR are noted (mT m^−1^, T m^−1^ s^−1^) for different gradient coils.

The Impulse gradient coil encoded higher spatial resolution in EPI by means of faster signal readout with shorter ESs, which reduces T2* signal decay and blurring (point spread function, PSF) on the image phase-encoded axis. PSF is smaller for the Impulse gradient coil when compared to the head-only AC84 and whole-body SC72 gradient coils, due to the shorter achievable ES and resultant echo train lengths (Fig. 3d). Deviations in the curves are due to forbidden frequency bands that avoid mechanical resonances by restricting minimum ES, affecting the dependent echo train length and T2* dependent blurring. Beyond a certain point, further increases in nominal resolution actually lead to an increase in PSF due to the extended echo train length. For each gradient coil performance, this inflection point is at a different resolution, with the Impulse gradient coil able to go to a higher resolution (roughly 0.5 mm) without an increase in PSF.

**Extended Data Fig. 3 ∣:**
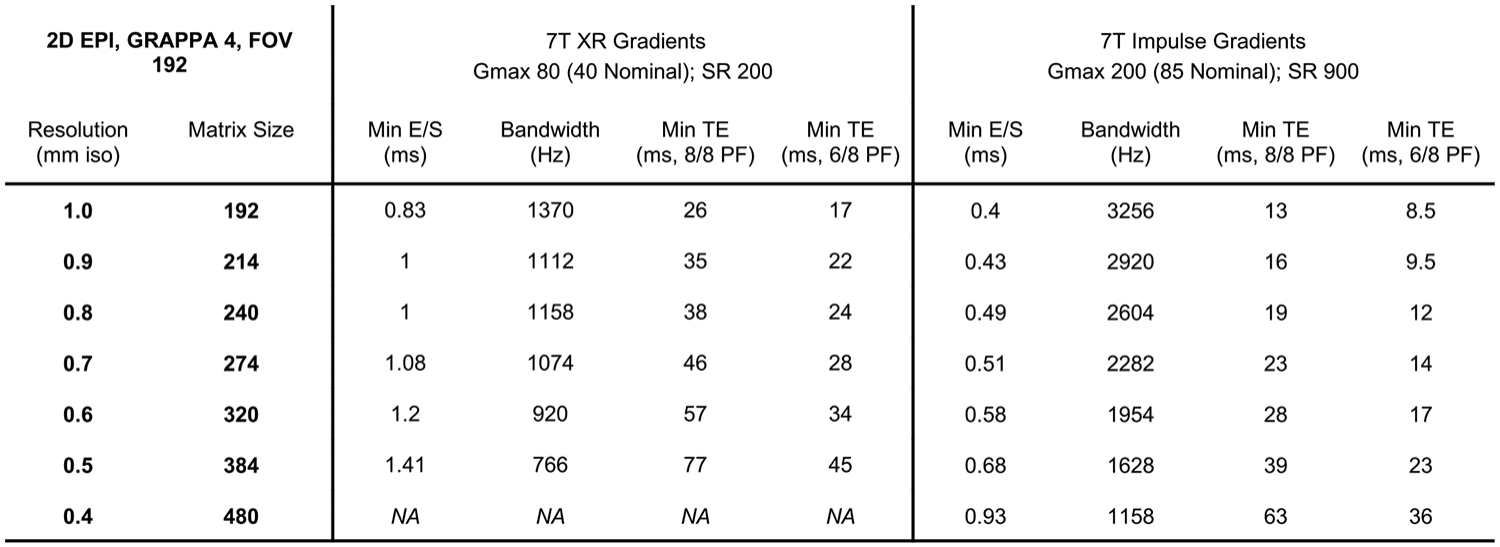
Gradient coil performance. Table of achievable resolutions with corresponding echo time, echo spacing and bandwidth for two different gradient coil performances.

**Extended Data Fig. 4 ∣:**
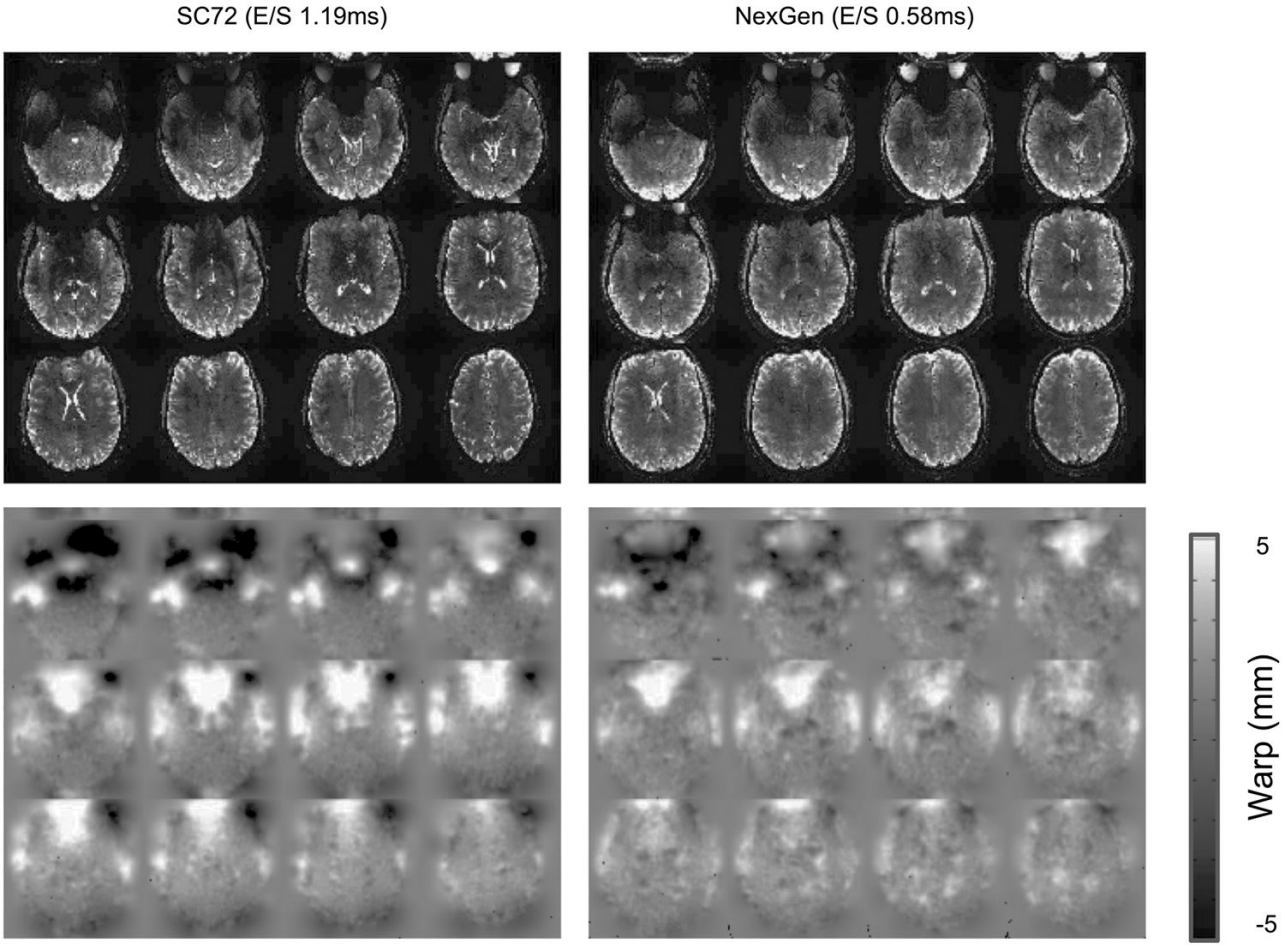
Gradient coil performance and distortions in EPI. Measures of distortion in high-resolution EPI using two different gradient coil performances. Distortion is measured as the level of non-linear warping required to bring two images with opposite phase encode (showing equal but opposite levels of distortion) into alignment. The distortion is expressed as a warp field map, showing the amount of distortion in mm experienced at each voxel, with the sign of the map value showing the direction of distortion along an image axis. Values near zero show little distortion, with more positive or negative values indicating greater distortion..

In addition to reducing the PSF, the advantage of the Impulse gradient coil to reach higher resolution EPI is the higher signal afforded by encoding a shorter echo time (TE). At 0.6 mm isotropic resolution, the Impulse gradient coil achieves a minimum ES of 0.58 ms reduced from 1.21 ms using the body gradient coil for a corresponding reduction of minimum TE of 17 ms reduced from 34 ms. To reach higher resolution of 0.5 mm, larger gradient pulses are required and the ES increases to 0.68 ms reduced from 1.41 ms and the corresponding TE is 23 ms reduced from 45 ms with the whole-body gradient coil with the latter unusable due to roughly 40% signal loss; Extended Data Fig. 3). The shorter ES also directly reduces image geometric distortion (Extended Data Fig. 4).

**Extended Data Fig. 5 ∣:**
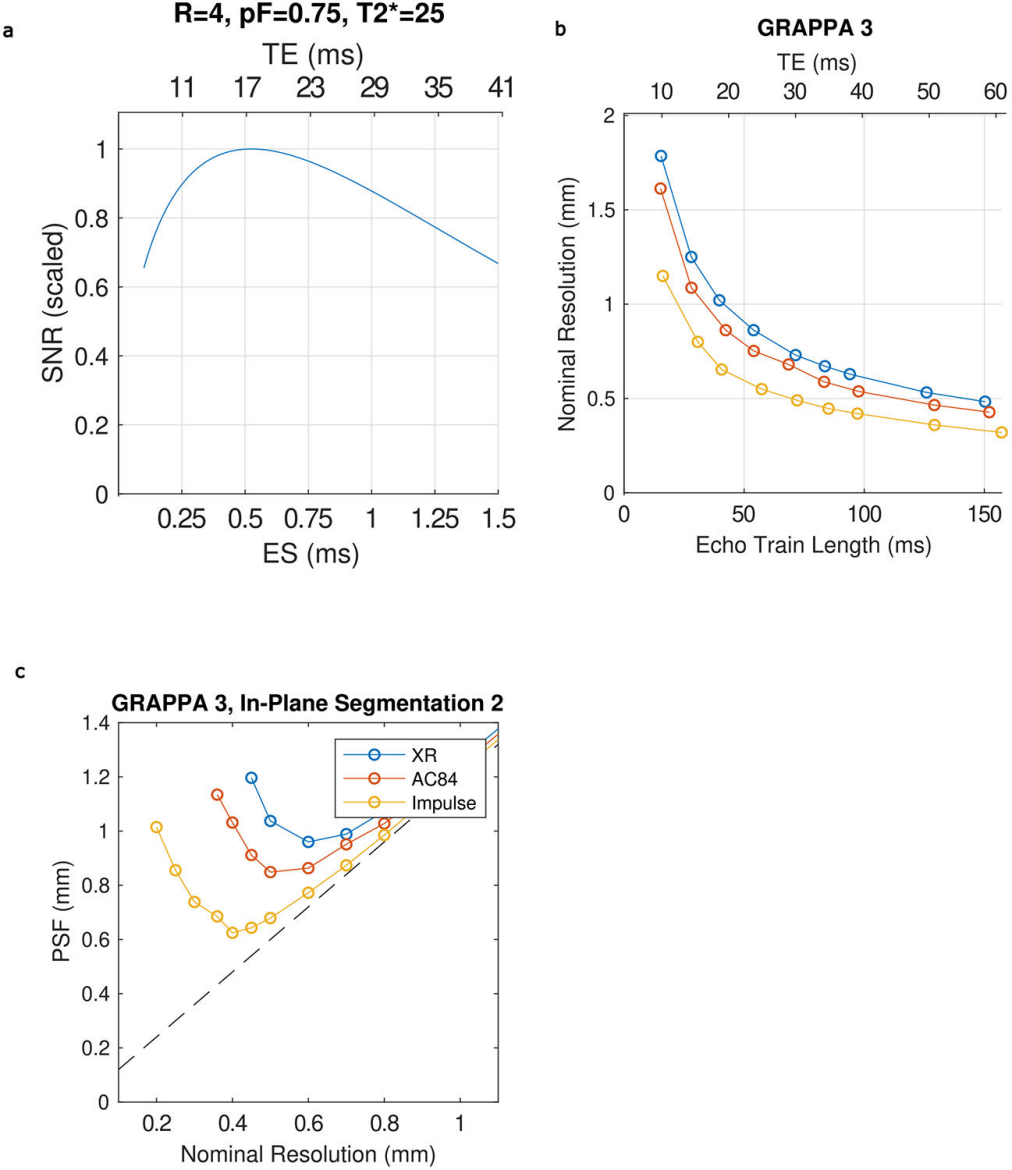
Gradient coil performance and EPI. (a), Simulation of the effects of Echo Time (TE) and echo spacing on SNR in EPI. (b), achievable nominal resolution for different minimum TEs for three different gradient coil performances when using GRAPPA acceleration of 4. (c), Point Spread Function (PSF) on image phase encoded axis (FWHM of Fourier transform of the modulation transfer function associated with T2* decay across the image readout, modeled using a T2* of 25 ms) versus nominal resolution for 3 different gradient coil performances for GRAPPA 3 combined with In-plane Segmentation (for an equivalent acceleration of 6). Dashed black line shows minimum achievable PSF for a given nominal resolution assuming no T2* blurring. (d) Comparison of SNR for central (blue) and peripheral (red) regions of the brain. Gmax, SR (mT/m, T/m/s) XR and SC72 whole-body gradient (80,200), AC84 head only gradient (80,400), Impulse head-only gradient (200, 900).

We also compared the achievable resolution using different gradient coil performances with very similar echo train duration using constant TE, FOV and number of phase encode lines. In this comparison, there was similar T2* decay and the relative PSF is constant in units of number of voxels (Fig. 3e and Extended Data Fig. 5b). For the three gradient coils (Impulse, AC84 head-only and standard SC72 whole-body gradient) the achievable resolution is 0.45, 0.61 and 0.7 mm, respectively (Fig. 3f). The respective achievable isotropic volumetric resolutions (voxel volume) are 0.09, 0.23 and 0.343 μl, corresponding to a 2.55- and 3.76-fold gain in volumetric resolution using the Impulse gradient coil.

### Neuroimaging evaluations

**Fig. 4 ∣:**
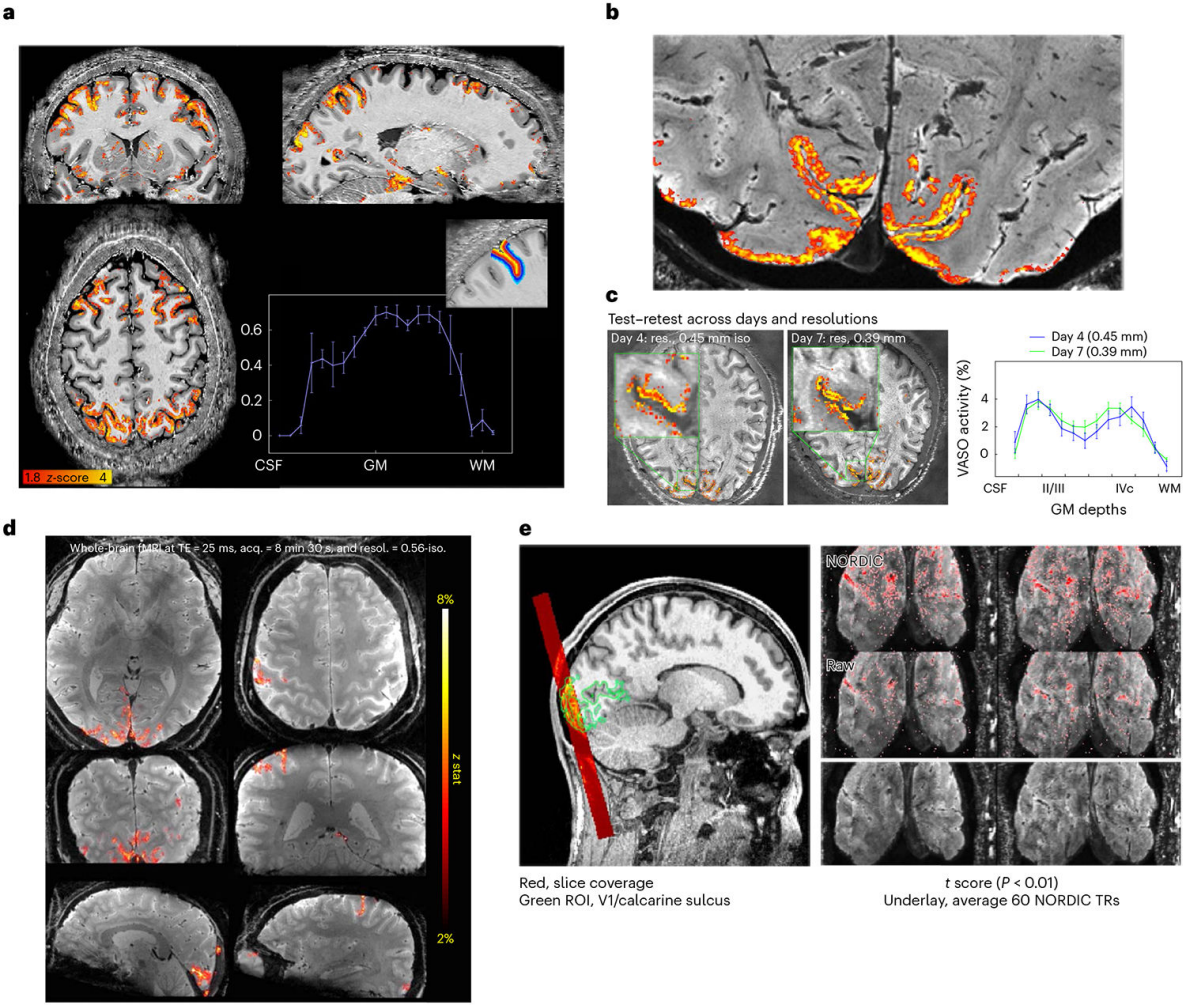
VASO and BOLD 3D EPI. **a**, Whole-brain VASO layer fMRI acquired at 0.64 mm resolution. A seed-based correlation map from a video watching task after layer-based smoothing displayed on the temporally averaged T1-weighted VASO volume. Activity across the gray matter ribbon (from CSF to white matter (WM)) is plotted with corresponding layer profiles displayed in the inset. Error bars refer to the variance of the signal within each layer across estimated columnar units spanning across approximately 30 mm of cortical ribbon of the sulci depicted in the layer mask. Adapted from ref. 44. **b**, Layer fMRI combining 0.45 mm and 0.39 mm isotropic resolution data (in 2-mm-thick V1 human cortex) differentiates activation in cortical layers (double stripes of activity) in supra- and infra-granular layers from a flashing checkerboard task. Activations overlaid on high-resolution GRE anatomical image. **c**, Test–retest of layer fMRI results in V1 across resolutions and days. Error bars defined as for **a**, across approximately 8 mm of cortical ribbon in the calcarine sulcus. Adapted from ref. 45. **d**, BOLD fMRI acquired (acq.) with whole-brain coverage at 0.56 mm isotropic (iso.) resolution (resol.) using 3D EPI with random *k*-space sampling scheme^35,36^ to increase SNR. **e**, BOLD fMRI with 3D EPI at ultra-high resolution acquired at 0.35 mm isotropic resolution, imaging visual cortex using stimulation checkerboard for 20 min. Activation maps thresholded at *P* < 0.01 (one side, no correction for multiple comparisons). NORDIC denoising was applied^59^. Adapted from ref. 38.

In comparison to BOLD-weighted fMRI images of equivalent resolution obtained on a standard scanner, the EPI images from the NexGen scanner show increased SNR and decreased g-factor noise (Fig. 3). The NexGen images also showed significantly increased temporal SNR (tSNR) (paired sample *t*-test, *P* < 0.001) in the brain periphery using the 64-ch Rx coil when compared to a standard 32-ch Rx coil (Fig. 3c, showing a mean peripheral tSNR of 9.82 versus 7.58 for the 32-ch Rx coil). For very high resolutions, three-dimensional (3D) EPI sequences^33-36^ have demonstrated higher SNR^37^ and improved slice profiles^33^ whereas thin slices are more difficult to define with two-dimensional (2D) selective excitation. 3D EPI sequences also allow additional flexibility in terms of using partitioned *k*-space data acquisition (segmentation) and/or parallel imaging to reduce effective ES and TE of readout times, allowing T2* decay, SNR and blurring penalties to be reduced. 3D imaging on the NexGen 7 T performed whole-brain coverage for fMRI with up to 0.56 mm resolution (Fig. 4d) and has allowed us to image at 0.35 mm resolution^38^ when using a reduced FOV (Fig. 4e), that is, using approximately 12-fold smaller voxel volumes relative to previous standard high resolutions of 0.8 mm achieved within acceptable scan times.

**Extended Data Fig. 6 ∣:**
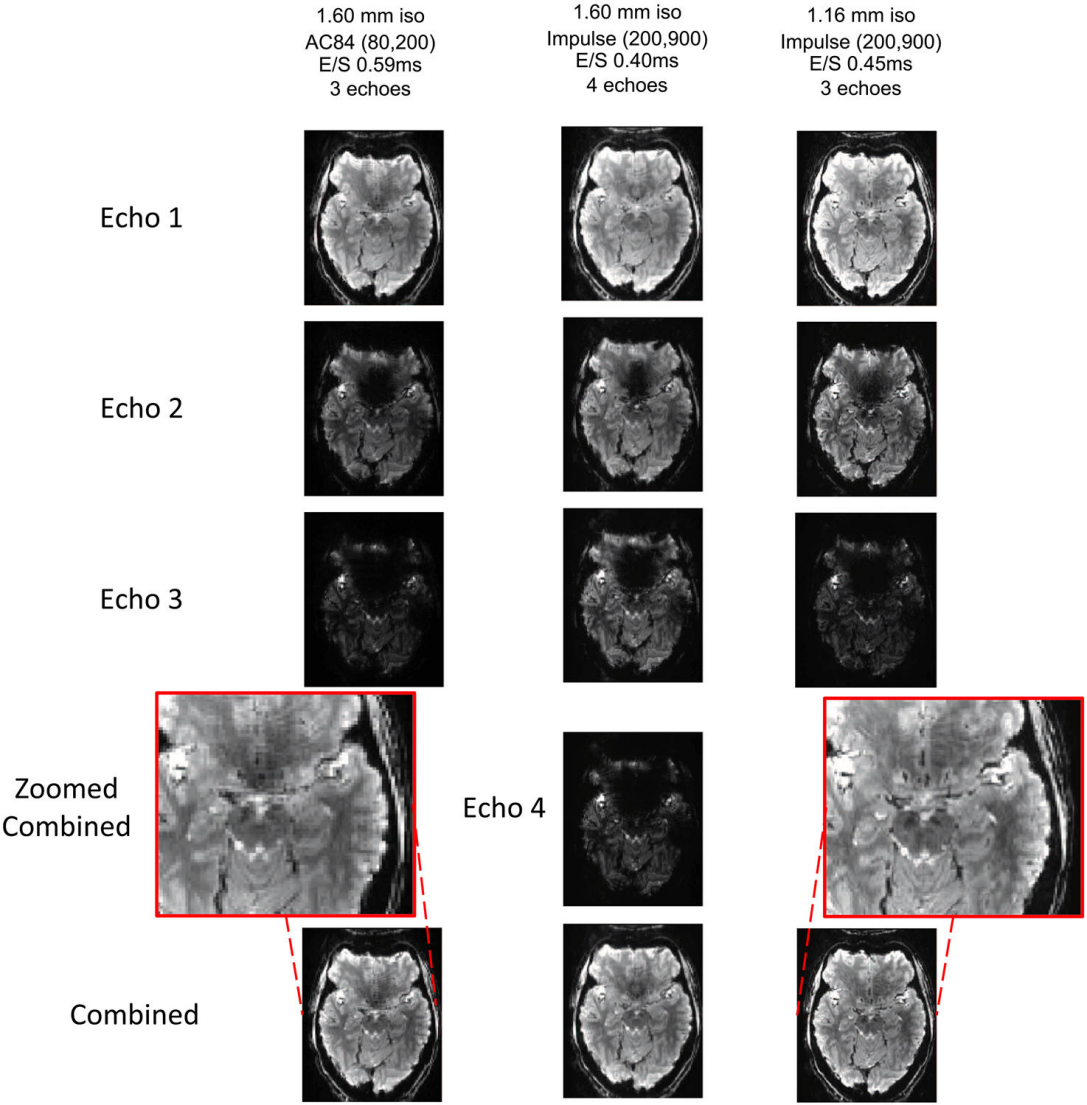
Multi-echo EPI imaging. Early TE of initial echo reduces susceptibility drop out of signal in optimally combined images. When compared to standard gradient performance, the Impulse gradient coil allows either an increase in resolution at equivalent TEs (1.16 mm isotropic versus 1.6 mm isotropic), or an earlier TE for the first echo and one additional echo in the same overall acquisition window (4 echoes versus 3 echoes).

Multiecho EPI is of value to fMRI studies, given that it can be combined across TEs to maximize tSNR and reduce signal dropout^39,40^, and can also be used to differentiate BOLD signal from noise components in fMRI time series using independent component analysis^41^. By shortening the EPI echo trains and ES on the NexGen 7 T scanner, multiecho EPI can provide resolutions not previously achievable (for example, 1.16 mm isotropic compared to the reported 2.0–2.5 mm resolutions^42^) or, alternatively, enable acquisition of four rather than three echo images at different TEs by comparison to a standard 7 T system (Extended Data Fig. 6).

**Extended Data Fig. 7 ∣:**
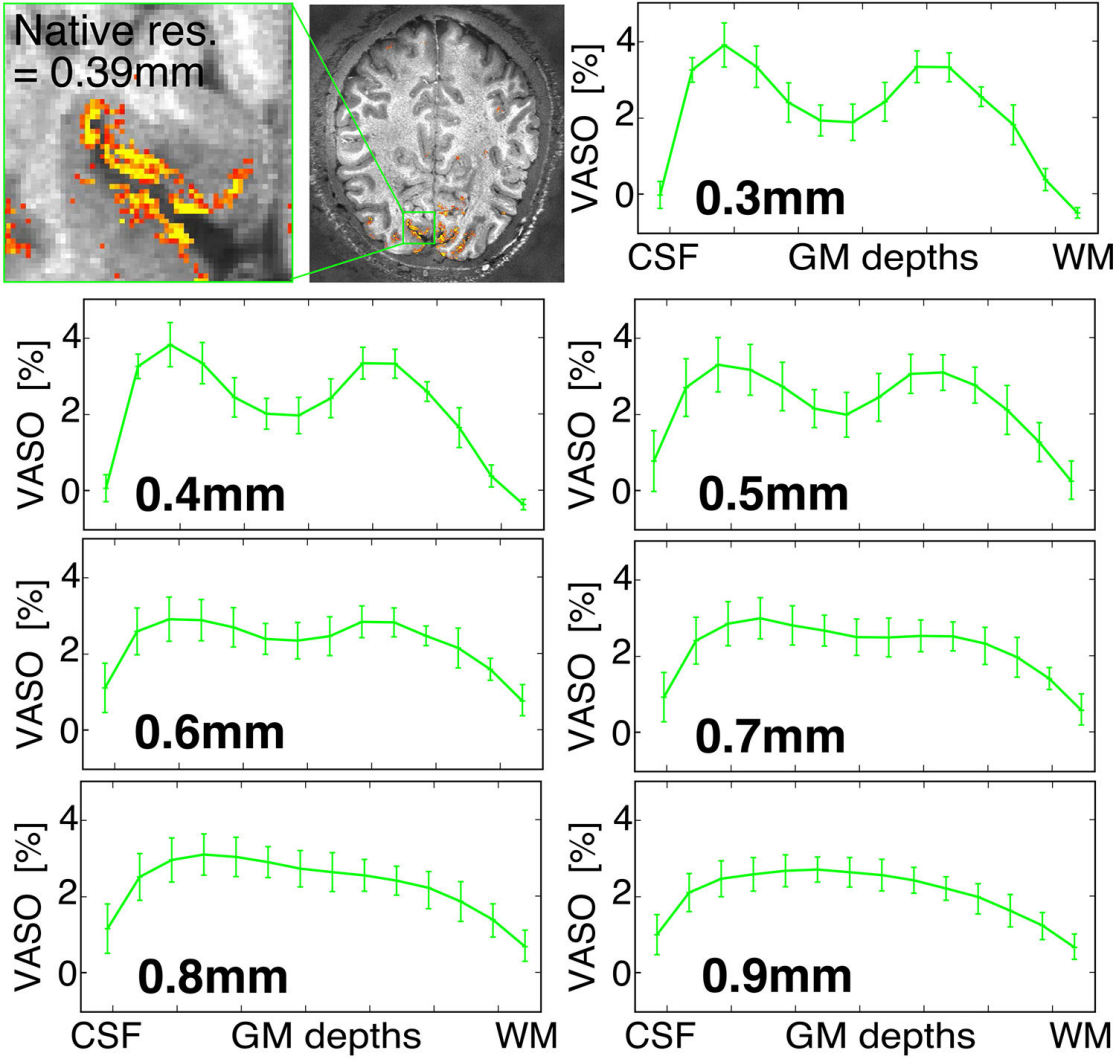
Effect of resolution on resolving laminar fMRI. Results down-sampled from acquired 0.39 mm isotropic voxels to lower resolutions.

Cerebral blood volume (CBV) contrast imaging using vascular space occupancy (VASO)^43^ pulse sequences, which localizes activation to specific cortical layers more precisely than BOLD by minimizing sensitivity to cortical draining veins, has shown depth-dependent activations in motor, somatosensory and frontal regions of cortex^13,14,16^ (Fig. 4). Using 3D segmented EPI^34^ with the hardware advantages of the NexGen 7 T system, we can currently achieve resolutions of 0.64 mm isotropic VASO images over the entire cerebral cortex^44^ (Fig. 4a), and 0.39 mm isotropic VASO images over slabs of smaller volume^45^ (Fig. 4b,c). Activity across the gray matter ribbon (from outer cortex to white matter) shows indications of sublayer peaks (Fig. 4a). Using VASO, we were also able to differentiate layer-specific activations in one of the thinnest areas of human neocortex (visual area V1, roughly 2 mm cortical thickness) that are unresolvable using the roughly 0.8 mm protocols achievable on conventional 7 T scanners (Fig. 4b and Extended Data Fig. 7).

**Fig. 5 ∣:**
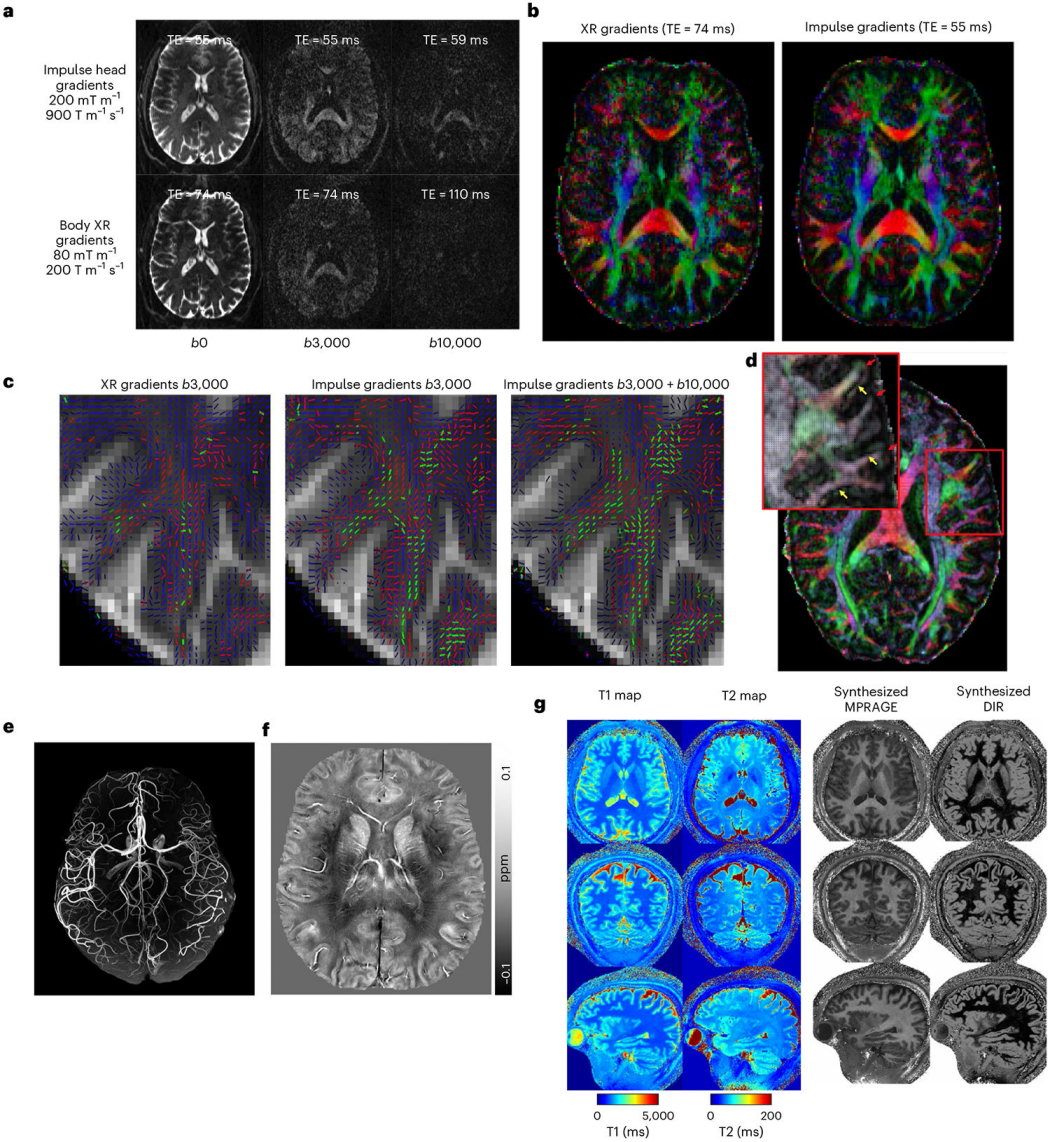
Diffusion and structural imaging. **a**, Diffusion-weighted MRI at various *b* values. Comparing images acquired with NexGen 7 T gradient coil performance in the top row to conventional gradient coil performance (80 mT m^−1^, 200 T m^−1^ s^−1^) in the bottom row. **b**, Improvement in color principal fiber orientation maps with shorter TE and higher SNR using NexGen 7 T gradient coil performance. **c**, Improvement in crossing fiber detection in complex white matter regions: primary (blue), secondary (red) and tertiary (green) fiber crossings in centrum semiovale white matter. The vector color intensities are modulated by the fiber’s respective volume fraction. Only fibers with volume fractions greater than 5% are shown. **d**, Pushing the spatial resolution to 0.8 mm using NexGen 7 T gradient coil. The primary diffusion direction map is overlaid onto fractional anisotropy for this 0.8 mm data. Yellow arrows indicate where white matter tracts turn sharply into the cortex and red arrows denote gyral crowns where the white matter tracts continue straight into the gray matter. Adapted from ref. 60. **e**, Time-of-flight 3D MRA at 0.4 mm isotropic. **f**, 3D QSM with tenfold acceleration. QSM map of a representative axial slice with a resolution of 0.21 × 0.21 × 1.5 mm^3^, reconstructed using the software STI Suite (UC Berkeley). **g**, Whole-brain quantitative mapping and synthesized images using MR fingerprint spiral imaging provided multiple image contrasts at 0.56 mm isotropic in a 4-min acquisition time. From left to right: acquired whole-brain T1 and T2 maps and several derived 3D image sets with different contrasts. MPRAGE (magnetization prepared-rapid GRE), double inversion recovery (DIR).

In diffusion imaging, the SNR advantages of the Impulse gradient coil are apparent even in single TR (relaxation time), unaveraged diffusion-weighted images (Fig. 5a) and in the color principal fiber orientation maps (Fig. 5b). The SNR and angular resolution advantage of the NexGen scanner resulted in up to 4.5-fold more detected tertiary fiber crossings (green vectors in Fig. 5c). The ability to increase spatial resolution in diffusion imaging to 0.8 mm on the NexGen scanner revealed sharply turning axons entering the cortex manifested as dark bands of fractional anisotropy, not typically seen at lower resolutions^46^ (Fig. 5d). SNR improvements are due to the faster diffusion encoding times and shorter TEs (59 versus 110 ms for *b* = 10,000 s per mm^2^) afforded by the Impulse gradient coil and are consistent with the up to threefold SNR gain as calculated by: exp(TE1/T2)/exp(TE2/T2), using a white matter T2 of 46 ms (ref. 47).

**Extended Data Fig. 8 ∣:**
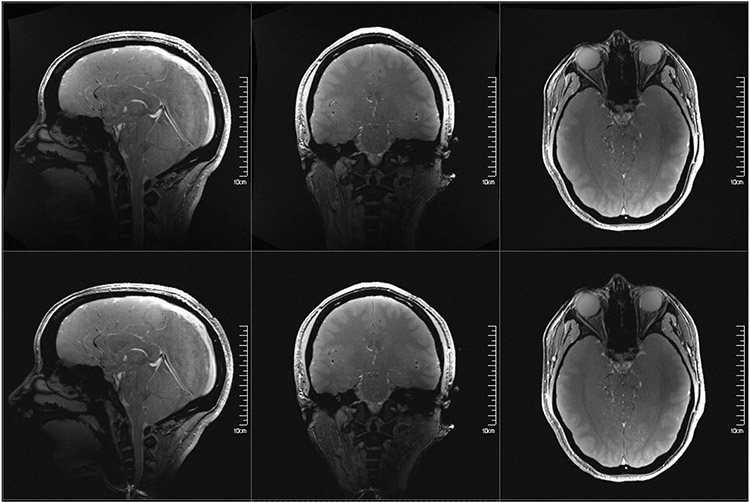
Head coverage of Impulse gradient coil. Shown in 3 perpendicular image planes with FLASH images, with and without distortion correction.

High-resolution anatomical brain imaging techniques also perform well on the NexGen 7 T (Fig. 5e-g). MR angiography (MRA) acquired at 0.4 mm isotropic resolution enabled excellent visualization of the peripheral branches in the anterior and posterior cerebral circulations (Fig. 5e). We also acquired a quantitative susceptibility map (QSM) based on 0.25 mm multiecho 3D T2* gradient echo (GRE) imaging (Fig. 5f). Diamagnetic susceptibility (negative values) shows myelinated white matter while paramagnetic susceptibility (positive values) shows iron rich regions and venous structures. A fine layer of iron rich region is observed at the frontal gray and white matter boundaries. For 3D QSM, the 96-ch array allows tenfold GRAPPA (generalized autocalibrating partial parallel acquisition) acceleration. Shortening the TE in 3D GRE sequences and the use of higher GRAPPA accelerations improved image quality and reduced the acquisition time of high-resolution imaging. The MR fingerprint technique simultaneously recorded images with different contrasts at 0.56 mm resolution using fast spiral non-Cartesian sampling trajectories^48^ (Fig. 5g): quantitative T1 and T2 maps, derived magnetization prepared-rapid GRE and double inversion recovery images. The entire set of multicontrast images were acquired in 4 minutes; with further calibrations and use of the full gradient coil performance, the data could be acquired in 2 minutes. Finally, we showed with T2*-weighted GRE anatomical imaging that the gradient coil coverage extends to the neck region (Extended Data Fig. 8). Although there is visible nonlinearity in the neck region along the *z* axis gradient, this is reduced by online distortion correction. Overall, we have demonstrated benefits of the NexGen 7 T for multiple anatomical MRI measures in addition to fMRI and diffusion imaging.

## Discussion

The resolution gains in MRI on the NexGen 7 T scanner using EPI with whole-brain coverage are volumetrically up to roughly 20 times higher compared to common 7 T fMRI resolutions 0.175 versus 4.1 μl for 0.56 versus 1.6 mm isotropic voxels^49^). The volumetric resolution gains for zoomed targeted fMRI imaging are on the order of ten times higher (0.059 μl versus 0.512 μl for 0.35 mm versus 0.8 mm isotropic voxels) with the actual gains higher due to PSF differences. Furthermore, such high-resolution fMRI imaging is not possible with conventional gradient performance since the signal decays before it can be read out, especially in brain regions with higher susceptibility causing faster T2* signal decay. Although the TE can be shortened by partial Fourier^50^, in-plane segmentation and higher parallel imaging acceleration^51,52^, these techniques typically involve trade-offs between SNR and PSF^32^ (Extended Data Fig. 5c).

Using 3D VASO imaging for fMRI with CBV contrast (rather than BOLD) at 0.39 to 0.45 mm isotropic resolution showed reproducible cortical layer-dependent functional activity, revealing laminar functional organization in the brain. Due to the Nyquist theorem, conventional 0.8 mm resolutions cannot reliably sample the individual layers of interest in thinner cortical areas (such as visual cortex area V1). Ultra-high-resolution CBV-sensitive imaging on the NexGen scanner has overcome a resolution barrier in human fMRI by differentiating activity of layers II/III and layers IVc, assumed to be the feedback and feedforward circuitry, respectively^53^.

The NexGen 7 T scanner has thus achieved an important milestone by extending fMRI studies to the mesoscale below 0.1 μl voxel volume. Information processing with network analysis tools^1^ to study interactions across many areas of the entire brain is now possible. The fMRI responses could be localized to the fundamental modules of brain computation across cortical depth, allowing investigation of brain circuitry toward a firmer basis for modeling of neurocircuitry than the temporal correlation studies so far used in fMRI connectomics.

In diffusion imaging, combination of higher SNR at 7 T with achievable higher amplitude gradients creates a platform for additional neuroscientific explorations. Gradient coils specifically designed for diffusion imaging can reach higher Gmax on 3 T scanners^22,24^; however, the Impulse gradient is operational at 7 T with the advantage of higher signal at the higher field strength. Future studies with coregistration of structural and functional data in single participants combining high angular resolution of fiber tracks and laminar fMRI of the whole brain should be possible for multimodal studies of the human brain.

It is possible to disseminate this NexGen 7 T technology to many currently installed 7 T scanners in neuroimaging centers. Proposed smaller 7 T magnets may also be realized^54^. Future work will be needed to address the data storage and processing needs arising from the larger size of raw data that is increased with resolution and with the larger number of simultaneous receiver channels. Higher magnetic field scanners (9.4, 10.5 and 11.7 T and in the future possibly 14 T)^55-58^ raise SNR in human brain imaging; however, fMRI and structural neuroscience studies are more difficult to conduct at these ultra-high field strengths due to the complexity of imaging with greater RF heating restrictions (specific absorption rate, SAR), B1/B0 magnetic field inhomogeneity and shortened T2* decay. Additionally, these scanners are at much higher cost. In closing, the numerous innovations developed and incorporated into the NexGen 7 T scanner will make diverse human neuroscience studies at ultra-high resolution routinely possible, including functional imaging of cortical layer and columnar organization.

## Methods

The NexGen 7 T scanner is based on a commercial 7 T scanner (MAGNETOM Terra, Siemens Healthineers) delivered to UC Berkeley and integrated onsite with our developed hardware systems and operating software. The scanner was developed through an academic-industrial collaboration between UC Berkeley, Siemens Healthineers and Siemens Medical Solutions, UCSF, Harvard-MGH, Advanced MRI Technologies LLC, CA, USA and MR CoilTech Limited, Glasgow, UK.

### Gradient coil design with balanced three layers

Shielded gradient coils are normally designed with an inner winding and a second, more distant shielding winding with counter current to cancel the external stray field at the surfaces of the magnet cryostat^61^. This effectively reduces eddy currents in the magnet cryostat. Head-only gradient coils^22-24,62^ with their smaller diameters and corresponding lower inductance can reach higher SRs than whole-body gradient coils; however, the usable SR and gradient performance is limited by physiological nerve stimulation.

The asymmetrical head gradient coil designed for our scanner, named Impulse (Siemens Healthcare), included a third intermediate winding layer of the saddle shaped coils^29^ to provide additional degrees of freedom for reducing mechanical torques, manipulate the concomitant field pattern that contributed to minimize PNS and allow use of higher SRs. While the basic wire-pattern optimization was achieved with boundary element stream function methods^63^, the design process additionally used combined electromagnetic and neurodynamic modeling^28^ in a detailed body model with a nerve atlas^64,65^. This allowed balance of PNS between the head and body regions, specifically shoulder and face areas, and was key to achieving winding optimizations that yielded substantially improved PNS thresholds and thus increased usable gradient coil performance (Fig. 1e). The gradient coil’s three-step asymmetric geometry (Fig. 1f) gives additional degrees of freedom to tune mechanical dynamics to minimize mechanical vibrations and acoustic resonances.

The gradient coil’s 44 cm inner diameter (Fig. 1f) was designed to leave space for efficient acoustic mitigation material and provide as much space as possible for larger RF coil arrays. The accessible bore space for the head and receiver–transmit arrays was 39 cm in diameter. The shoulder space is the final of the three steps in the gradient coil-space and is contiguous with the 60 cm diameter magnet space allowing unrestricted participant access to reduce claustrophobic feelings.

The Impulse gradient coil was designed for high gradient linearity (less than 10% on a 20 cm (diameter sphere volume), a relatively large inner coil diameter (44 cm) and high amplitude and SR (up to 200 mT m^−1^ and 900T m^−1^ s^−1^ per axis). Using a gradient power amplifier producing 1,200 A, 2,250 V on each axis, the Gmax achievable is 200 mT m^−1^ on each of the three axes.

With increased gradient pulse amplitudes and high PNS thresholds, the Impulse head gradient coil can refocus echoes faster, which is key to encoding higher resolution in EPI (for example, for functional brain imaging) (Fig. 3a). Short ES reduces the total time to read out the echo train and thus shortens the achievable minimum TE for a given resolution, yielding exponentially increased SNR (albeit with a smaller concurrent SNR loss from higher square root of signal bandwidth increase) (Extended Data Fig. 5a). When the in-plane FOV is held constant, the PSF (in mm) initially decreases as the nominal resolution is increased due to the decrease in voxel size.

#### PNS supervision.

The supervision and limitation of PNS during scanning are implemented identically as with the standard MAGNETOM Terra system’s stimulation monitor (Siemens Healthcare). PNS limits have been adapted for the Impulse gradient coil based on the stimulation approximation by filtering and evaluation (SAFE) model^31^. The SAFE model for the Impulse gradient coil used the average of individual stimulation thresholds of 33 test participants reported in a different paper^27^ in a study conducted at the Siemens factory in Erlangen, Germany, under ethics approval and with written informed consent. The SAFE stimulation limit is derived from the experimental value of the volunteers’ stimulation level in a model that filters and rectifies the signal and then weighs with scale factors to approximate the stimulation signal and determine Stimlim as the upper limit of the first level controlled operating mode. The SAFE stimulation threshold is given according to International Electrotechnical Commission (IEC) standard IEC 60601-2-33 by 0.8 × Stimlim and limits the scanner’s normal mode of operation.

Based on the statistical distribution, it can be expected that up to 50% of all patients will experience at least mild stimulations when reaching this stimulation limit in first level mode, according to IEC standard 60601-2-33 (2010, 3rd edn), clause 201.12.4.102. The applied SAFE model (solid red lines in Fig. 1e) limits the gradient pulses conservatively below the results of the study, thus ensuring safe operation of the gradient coil in nearly all instances.

#### Gradient cooling system.

Going from the 39 cm inner diameter of the AC84 gradient coil to the larger 44 cm diameter of the Impulse allowed for the integration of more complex RF transmit and receiver hardware with larger arrays. However, this increased the gradient coil impedance and required a larger driving current of 1,200 A from the gradient power amplifier. When operating with such high current density, it is necessary to efficiently eliminate more than 20 kW of heat dissipated by the electrical power. To meet this specification, a cooling system was designed by Stocker and Dietz^66^ using a multifilament copper conductor directly cooled by stainless-steel tubing carrying the cooling water (Fig. 1g). The stainless-steel tube has lower conductivity than the surrounding copper filaments, giving lower sensitivity to eddy currents. The steel has negligible magnetic properties, thus minimally perturbing the homogeneity of the B0 magnetic field. The large wire cross-sections, short cooling loop lengths (using parallel water circuits) and high flow rates (enabled by the high pressure stainless-steel tubing) facilitated sufficient heat removal and maintained low gradient coil temperatures, as monitored by more than 50 temperature sensors. Maximum temperature measured on the peak hot spot inside gradient cooling increased from 20.5 to 31.7 °C at 150% duty cycle, 80 mT m^−1^ for 30 min.

#### Shim coils and RF shielding.

Active resistive shim coils were incorporated into the gradient coil to include first- and second-order harmonics. To minimize eddy-current induced heating in RF shielding from the high SRs of 900 T m^−1^ s^−1^, thinner copper was used for the RF shielding laminated on the inner surface of the gradient coil.

Several challenges in hardware integration arose during testing that had to be solved:

1. The acquisition computer had to be modified to handle the larger data size acquired with the 128-ch receiver system and larger matrix sizes used for high-resolution imaging, including increasing the disk space for raw *k*-space data on the measurement and reconstruction system (MaRS).
2. The electromagnetic fields inside the scanner can create strong eddy-current induced forces and mechanical vibrations in RF receiver hardware. Eddy-current induced heating of the external RF shield of the Tx coil (MR CoilTech) and spike artifacts during the EPI acquisitions were solved with onsite coil work to shorten the shield away from the rear of the gradient coil where eddy currents were most severe.
3. Mechanical resonances were encountered that led to cracks in the head gradient coil’s anterior support structure, and were overcome initially by excluding specific ESs. This was later fully resolved by a different gradient coil support structure that was designed, fabricated and tested by Siemens scientists.
4. Ghosting artifacts in EPI were greatly reduced by improved compensation of short-term eddy-current terms in pre-emphasis gradient waveforms while maintaining negligible long term eddy currents. Additional reduction in ghosting artifacts was achieved by adjusting the timing and shape of readaxis dephasing pulses before the collection of phase navigator echoes and image echoes. Concomitant gradient compensation was performed via modeling using existing approaches^67^.

#### Mechanical resonances.

During initial testing of EPI sequences, specific mechanical resonance peaks caused cracks in the anterior edge of the extended support on the gradient coil’s longitudinal axis that were more than 40 cm from electrical wire windings, which were unaffected. Further damage was avoided by prohibiting the specific ESs corresponding to specific mechanical resonance frequencies. The gradient coil was operated for 5 months until a second gradient coil was fabricated with a modified support structure to eliminate the unwanted mechanical resonances. The replacement gradient coil was installed in the scanner at the end of 2021 and has been fully operating without the problem recurring. The second gradient coil underwent no changes in wiring pattern; consequently there were no changes in force, torque balance or PNS thresholds. Noting that almost all MRI gradient coils have specific forbidden frequency bands, the Impulse gradient coil was designed to allow the most useful bandwidth EPI readouts for high-resolution brain imaging, with forbidden ESs between 0.82 and 0.92, 0.46 and 0.48, and 0.35 and 0.36 and at 0.26 ms corresponding to Eigen-frequencies in ranges (540–610, 1,025–1,095, 1,375–1,445 and 1,860–1,980 Hz) of the Impulse gradient coil on the *x* axis, *y* axis and the combined *x* and *y* gradient axes. Pulse sequences generating lower mechanical frequencies (that is, GRE) do not require this restriction.

To determine a worst-case level of acoustic noise generated by the operation of the Impulse gradient coil, a series of acoustic noise measurements was performed using a Bruel and Kjaer 2260 Observer sound level meter. A spectrum of the acoustic noise was generated by pulsing all three gradients with the same amplitude in a pink noise frequency distribution encompassing the maximum possible frequency (determined by the rise time). The overall loudest peak was identified at a frequency of 552 Hz (equivalent to an ES of 0.92 ms). The timing parameters for a bipolar gradient waveform achieving this ES were calculated, and the maximum gradient that could be used within PNS limits was identified, corresponding to a Gmax of 98 mT m^−1^ and an SR 213 T m^−1^ s^−1^. Using this sequence sound levels reached a value of 120.6 dB(A) (Table 1), which is within the safety limits when using at least 28 dB attenuation earplugs.

Sound levels were also measured using a high-resolution EPI sequence using two gradient strengths typically used for neuroimaging studies, at a range of ESs using three orthogonal readout directions, and sound levels were within prescribed safety limits (less than 99 dB) when using 33 dB attenuation earplugs (Extended Data Fig. 1).

### RF transmit and receive array coils

The design and construction of the transmit and receive coils was challenging due to the tight space constraints imposed by the head gradient coil and the increased labor required for fabrication compared with smaller 32 channel arrays, and the increased difficulty of identifying circuitry failures in the closely packed circuits. Compared to 3 T coil arrays^25^, the development of high-density coil arrays at 7 T is substantially more complex due to the need for a local transmit coil. First, the receive array and its electronics must be packed within the transmit coil and, second, the interaction between the transmit and receive coils must be controlled to preserve the transmit efficiency and to ensure that the spatial distribution of the transmit B1 is not altered.

To increase the likelihood of obtaining an array with increased performance compared to the current industry-standard 32-ch 7 T head coil (32-ch Rx) and assess the benefit of higher channel counts, three different research groups built the RF coils for our project. MR CoilTech Limited built two RF receiver–transmit coils, used for all imaging on the NexGen 7 T scanner. Figure 2a shows the 96-ch Rx array combined with 16-ch transmit array (16-ch Tx, 96-ch Rx) coil, (Fig. 2a(i)-(iii)) and a 64-ch Rx array combined with an 8-ch transmit array (8-ch Tx, 64-ch Rx) (Fig. 2a(iv)-(vi)).

#### Transmit system and SAR management.

The scanner is equipped with a 16-ch parallel transmit (pTx) system capable of delivering 2 kW peak power per channel with independent RF waveform generators for fully dynamic pTx experiments. The scanner assures safe operation by monitoring the forward and reflected power on each channel and adheres to the IEC 60601-2-33 guidelines for global and local SAR^68^.

The SAR management is via a system for safe scanning by monitoring global and local SAR with an identical implementation of supervision software as the FDA 510(k)-approved MAGNETOM Terra, and by using virtual observation point (VOP) models specific to individual coil arrays for parallel transmit (pTx) operation. Before each measurement, ‘Look Ahead’ monitoring will calculate the worst-case SAR quantity values and compare them with the corresponding limit values as defined by IEC guidelines, providing parameter suggestions to reduce values if necessary. During a measurement, the online monitoring system constantly measures the transmit power and ensures that the IEC standard limit values are observed. Examinations in progress will be aborted if the limit is exceeded. The 16-ch transmit system also enables B1 shimming to yield a more uniform transmit RF field, allowing sequences that require this uniformity to operate optimally^69^.

In a pTx system, the SAR is a function of the superposition of the amplitude and phases applied to individual array elements. When the transmit arrays are driven in the circularly polarized mode, which has a fixed amplitude and phase relationship between the array coil elements, the forward power is controlled by a *k*-factor. This value is derived from the electromagnetic simulations and a safety factor of at least two is added to the worst-case local SAR. For pTx operation, a VOP file for each RF coil was generated^70^. Different body models were simulated and the final VOP file was generated by concatenating the datasets. A safety factor, overestimation factor and manufacturer-recommended error tolerance was applied to generate the final VOP file.

#### 16-ch transmit array.

A dual-row transmit array that offers 3D B1+ shimming capability was constructed (Fig. 2a(ii))^71,72^. The antenna extended 21 cm along the *z*-direction, and each array element consisted of ten capacitors to tune the loop to the Larmor frequency. Coupling between the adjacent elements in each row is canceled by geometric overlap and the diagonal elements between the two rows are decoupled by counter-wound inductors^73^. The loss mechanisms were investigated using 3D electromagnetic simulations to create an efficient design. To reduce radiation loss, a slotted double-layered 30-cm-long RF shield was placed concentric to the array elements with a 30 mm gap. In initial testing, the strips in the RF shield experienced eddy-current induced heating on the outer housing, which was produced predominantly at the location of maximum dB/dt, 40 cm off isocenter in the head direction. The heating was reduced to within 36° by shortening the length of the RF shield and narrowing the strip width.

#### 8-ch transmit array.

The 8-ch array was based on a nested transmit array design^74^, consisting of eight overlapped segmented loops (Fig. 2a(v)). Coupling between adjacent array elements was minimized by geometric overlap, and transformer decoupling was implemented to decouple the next-neighboring elements. The RF shield design was adapted from the 16-ch array design as it was already optimized to minimize gradient induced RF heating. In addition to minimizing radiation loss, the local shield improves robustness of RF coil tuning and makes the array less sensitive to its position on the patient table.

#### 96-ch receive array.

A single shell tight fit helmet (175 mm along left to right, 215 mm along anterior to posterior) was chosen to maintain sample loading on the small receive elements. The helmet was shaped using anthropometric data and had a curvature beneath the skull base to provide a comfortable fit. The 96 receive elements were arranged symmetrically in six rows. There are 12 elements in the top row, 18 in the second and 24 in the third row covering from the vertex of the dome to the level of the nose bridge. The other 42 elements covered the remainder of the helmet surface^72^. Adjacent elements within the row and between the rows were geometrically overlapped. The staggered arrangement in the top three rows resulted in strongly coupled diagonal elements and these were decoupled using transformer decoupling. The size of each receive element was about 35 × 45 mm. The two eye loops were larger. An elaborate test setup was established to bench-test the transmit and receive array in conjunction. This further allowed us to test and control the interaction between the 16-ch transmit array and the 96-ch receive array to ensure that the spatial distribution of the B1+ field was not altered due to the receive array. A picture of the completed receive array and the final assembly is shown in Fig. 2a,c.

#### Split-top 64-ch receive array.

A split-top sliding mechanism as in industry-standard 32-ch head RF coils was implemented within the tight space of the patient table to improve comfort while positioning the participant on the 8-ch Tx, 64-ch Rx coil. There are 24 elements in the anterior half and 40 elements in the posterior half, and the dimensions of the loop were about 45 × 55 mm. The receive elements are arranged in columns and there is an offset between the columns such that each element in one column symmetrically overlaps with two elements from the adjacent column. This offset was also implemented between the adjacent columns of the two halves, and the overlap is adjusted such that the mutual coupling cancels when the anterior half is moved to its final position after positioning the participant^75^. Each loop consisted of one high-Q variable capacitor and three fixed capacitors, and connected to a low impedance preamplifier (Wantcom Inc.). A shielded cable trap is connected between the coil input and the preamplifier. The internal dimensions of the receive helmet is 180 mm along the left to right direction and 215 mm along the anterior to posterior direction.

For both the 96-ch Rx and 64-ch Rx coils, all circuit boards were miniaturized and a detailed computer-aided design model was created to visualize component placement, cable routing and packaging. This was essential to optimally use the limited space and achieve the visual field needed to support fMRI studies.

#### Receiver–transmit array performance.

While image resolution and receive SNR are most often highlighted, the importance of transmit performance to achieve a usable RF coil setup gets submerged. Preserving the transmit performance is considerably more challenging while combining transmit coils with high-density receive arrays together with the space constraints of the head gradient coil insert. To minimize interactions with the transmit array, the receive circuit boards within the transmit field were oriented orthogonal to the transmit elements and the receive cables were routed along the virtual ground of the transmit elements. The S-parameters of the transmit array were fine-tuned in the presence of the actively detuned receive array. For both the transmit arrays, the reference voltage required to achieve 90° flip angle was in the range of 15 to 20% higher compared to the industry-standard 32-ch Rx and this is mainly due to the increased shielding effect of the high channel count receive arrays. However, the spatial distribution was preserved as evident from the images presented in this article that were acquired in circularly polarized excitation by applying the theoretical phase offsets.

As noted earlier, the 16-ch Tx, 96-ch Rx coil was built first and was used in initial stage development and troubleshooting. The 8-ch Tx, 64-ch Rx coil was developed later incorporating learnings from the 96-ch Rx coil. The unique RF configuration meant that these coil arrays could only be partially tested in a standard 7 T scanner during development. Elaborate RF coil test setups were developed at the coil development laboratory in Glasgow as well as onsite in Berkeley. The initial challenges were unique to the high-performance gradient system and included troubleshooting for spikes at high gradient strengths and gradient induced heating on the local RF shield of the transmit array. Overlapping receive coil conductors that are too close created spikes due to vibrations at high gradient strengths. The 64-ch Rx coil benefitted from the knowledge gained from the first array and incorporated further improvements such as reduced solder joints, low loss cable between the coil input and preamplifier as well as a custom-made high-Q trimmer capacitor in the loop. Hence, there is potential for further improvement of the 96-ch Rx coil array. A full characterization of the two RF coils is the subject of a separate article. After initial troubleshooting to eliminate image spike artifacts and gradient induced RF heating, the two coil arrays have been used regularly since January 2022 and have been functioning reliably. The coils needed remote service twice, which was traced to a snapped connector and a cold solder joint resulting in preamplifier failure.

#### Receiver system with system control computer.

**Extended Data Fig. 9 ∣:**
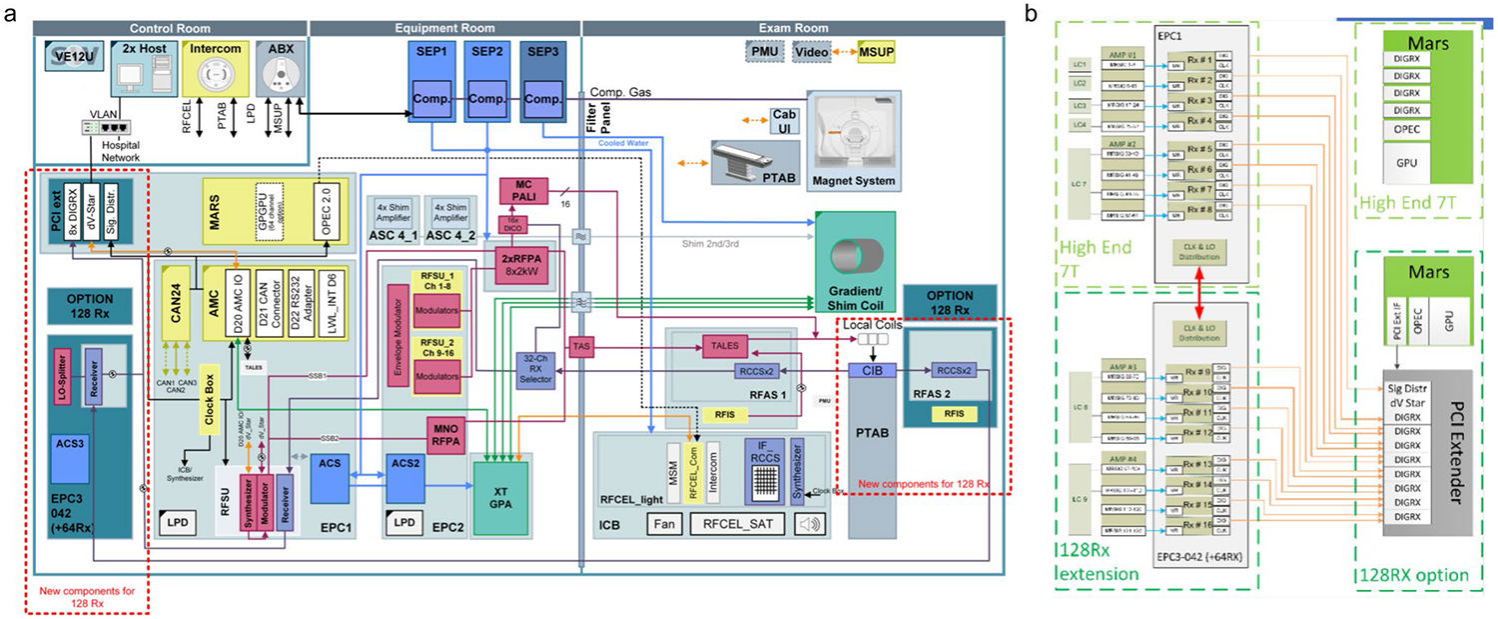
Hardware Developments for NexGen 7 T. a, Diagram overview of the whole scanner systems showing the Incorporated components of the 128-channel system. b, Diagrams of scanner’s measurement and acquisition (MaRS) computer showing adaptation for higher channel receiver.

Many MR receiver systems promote a large number of receive channels (for example, up to 228 in one 3 T scanner: Vida, Siemens), but these are the number of connectable channels, not the number of simultaneously active channels (which is limited to 32 or 64 channels). Up to 96-ch receiver coils have been used at 3 T and a simulation of 256-ch coil at 3 T has been proposed. For simultaneous acquisition of 128 receiver channels and supporting the use of 8, 16, 32, 64, 96 and 128-ch Rx head coils, the 7 T MR system was adapted from the existing 64-ch receiver system that is processed analogously by 8-ch Rx cassettes and fed digitally to four digital DIG-ch Rx cards on the MaRS. The extension to 128-ch Rx was required for the Berkeley NexGen 7 T project, comprising a duplication of the analog-ch Rx part (shown as EPC3) with another 8-ch Rx cassettes. As the number of PCI slots on the MaRS is limited, a PCI extender was needed to accommodate eight DIG-ch Rx boards (Extended Data Fig. 9).

The requirement for whole head FOV at high spatial resolution necessitated a higher sampling rate of signal readout with shorter dwell time than the standard scanner that uses 2 μs with oversampling. We therefore modified the receiver to achieve 0.5 μs dwell time for 1 μs with oversampling.

Many additional challenges were addressed for this project, such as the synchronous clock and local oscillator distribution on the analog Rx part, the design of a custom interface connector for 128 Rx channels and the digital data acquisition and throughput to the reconstruction computer (MaRS). The latter required improving the memory capacity of the reconstruction computer to superfast memory modules. Extended Data Fig. 9 shows a system diagram of the 7 T scanner with highlighted changes of receiver parts and components added for the 128-ch receiver system.

### Imaging parameters

MRI data were collected on the NexGen 7 T at UC Berkeley, and on a standard Siemens 7 T scanner for comparison at San Francisco Veterans Administration hospital. Data collection procedures were approved by the Institutional Review Board at UC Berkeley (CPHS no. 2020-07-13437) and UCSF/SFVA (IRB no. 16-20872). Written consent was collected from each patient and patients were compensated for their participation. A total of nine participants (three female, mean age 32.22 years, mean height 171.6 cm, mean weight 64.46 kg) were scanned across a range of different scan types for this study. Participants were monitored for any feelings of discomfort, including PNS, during all scans. Thus far, more than 100 individual participants have been scanned on the NexGen 7 T scanner with no adverse effects. A small number (around 5–10 participants) have reported some PNS, primarily felt as a pressure around the sinuses, when very short ESs are used (for example, 0.32 ms in a 1.6 mm isotropic multiecho EPI protocol, which uses a gradient amplitude of 58 mT m^−1^ and an SR of 832 mT m^−1^ ms^−1^). The scanner performance levels that led to PNS responses fell within the usable range set by the SAFE model used for scanner operation, but still might be expected to lead to PNS in a subset of participants. In instances where PNS occurred, protocols were amended for that participant to avoid PNS. Stimulus presentation was done using PsychoPy (v.2022.2.5).

#### SNR and g-factor comparisons.

The high-density receiver arrays of the NexGen scanner were compared to the industry-standard commercial (Nova Medical) 32-ch Rx, 8-ch Tx array coil provided on most 7 T scanners. The 32-ch coil did not fit into the NexGen scanner’s head gradient coil; therefore comparisons were made to the 32-ch coil in a conventional 7 T scanner by matching imaging parameters and using the same gradient settings. Receive SNR measurements used a whole-brain 2D proton-density weighted GRE sequence with a nominal flip angle of 90° to limit the impact of B1+ inhomogeneities on the signal intensity (TR, TE and flip angle of 5 s, 3.82 ms and 90°; slice of 2 mm; matrix of 256 × 88, FOV = 256 × 176 mm^2^; readout bandwidth of 335 Hz per pixel and TA = 7 min and 22 s).

Noise-covariance information was acquired using the same pulse sequence, but without RF excitation. Following the method of Kellmann et al.^76^, SNR maps used the noise-covariance-weighted optimal coil combination of the individual channel images, where the weights use coil sensitivity maps and noise-covariance information^76,77^. The excitation flip angle maps were acquired using a preconditioning saturation pulse with a turbo-fast low-angle shot readout^78^ (TR, TE and flip angle of 5 s, 2.02 ms and 90°; slice of 1.5 mm; matrix of 256 × 88; FOV = 256 × 128 mm^2^; readout bandwidth of 335 Hz per pixel and turbo factor of 128). The SNR maps were then normalized by dividing them by sin(flip angle) to isolate the receive sensitivity in the SNR maps. Figure 3c shows improvements in SNR across array coil size compared to using boxplots of flip angle-corrected SNR within a central and peripheral region of interest (ROI) for each array coil.

Retained g-factor maps (1/g) were computed for the combination of several in-plane accelerations (along the anterior–posterior phase encode direction) with different multiband acceleration factors, using coil sensitivity maps estimated from the fully sampled data using ESPIRiT^79^ and measured noise-covariance matrices^51^. To compare 1/g distributions across different array coils, boxplots of 1/g values across the brain were plotted across different accelerations for each array coil.

SNR and 1/g maps were compared to a commercial 32-ch coil^80^ on a conventional whole-body 7 T Terra system.

#### Gradient coil performance comparison.

The achievable resolution for a given TE and echo train time for three different gradient coil performance settings was calculated by setting a constant FOV (200 mm), setting the gradient coil performance limits from duty cycle limits during EPI time series acquisitions to those of three different gradient coils for EPI imaging: XR whole-body gradient coil (absolute Gmax 80 mT m^−1^, duty cycle limited nominal Gmax 40 mT m^−1^, SR 200), the AC84 head-only gradient coil insert (absolute Gmax 80 mT m^−1^, nominal Gmax 50 mT m^−1^, SR 333) and the high-performance Impulse gradient coil (absolute Gmax of 200 mT m^−1^, nominal Gmax 85 mT m^−1^, SR of 200 T m^−1^ s^−1^). While keeping TE constant, resolution and ES were optimized for the above three different gradient coil performance settings (X-ray whole-body gradient coil, AC84 head-only gradient coil, Impulse head gradient coil). For each gradient coil performance setting, resolution was increased until the achieved minimum TE matched a target TE. Estimates were compared for two different GRAPPA accelerations (three and four), with a constant partial Fourier factor (PF) of 0.75. In vivo tests of resolution were performed using a 2D EPI sequence (GRAPPA 4, PF 0.75 with projection onto convex set reconstruction, FOV 196 × 196 mm, 1 mm slice thickness, 1 mm slice gap, 4.5 s TR, 54 slices, 26 ms TE, adaptive combine coil combination). Gradient coil performance setting specific parameters were: X-ray whole-body gradient coil, matrix size 282 × 282, in-plane resolution 0.7 mm, ES 1.07 ms, bandwidth 1,045 Hz; AC84 head-only gradient coil, matrix size 320 × 320, in-plane resolution 0.61 mm, ES 0.94 ms, bandwidth 1,200 Hz; Impulse head gradient coil, matrix size 436 × 436, in-plane resolution 0.45 mm, ES 0.72 ms, bandwidth 1,640 Hz).

Blurring (PSF) on the image phase encode axis is highly dependent on the interaction between T2* decay, ES, acceleration and power factor. The dependence of PSF on gradient coil performance was defined and simulated as in previous work^32,81^ as the anisotropic spread of information in the image from an idealized point source. The full-width at half-maximum values of the simulated PSFs were calculated by taking the magnitude of the Fourier transform of the function reflecting the modulation of *k*-space data (signal reduction by T2* relaxation weighting at different positions in *k*-space), for which the magnitude is the modulation transfer function. The T2* weighting along an echo train and dependent truncation of *k*-space limits the effective resolution of the image.

#### Distortion comparisons.

The differences in distortion achieved for different gradient coil performances were measured by setting the performance limits to those of the SC72 and Impulse gradient coils. High-resolution EPI data (0.6 mm) were collected using the two performance levels, with both an anterior–posterior and posterior–anterior phase encoding direction (Extended Data Fig. 4). For each gradient performance setting, the distortions in the sets of images with opposite phase encoding should be equal but opposite. Nonlinear alignment (using AFNI 3dQwarp) of the two oppositely distorted images allows the level of distortion for that gradient coil performance to be calculated. The distortion is expressed as a warp field map, showing the amount of distortion in mm experienced at each voxel with the sign of the map value showing the direction of distortion along an image axis. Values near zero show little distortion, with more positive or negative values indicating greater distortion.

#### BOLD-based fMRI.

Initial implementation of EPI was hampered by incomplete eddy-current correction, which was greatly improved by recalibrating the gradient pre-emphasis to compensate for short-term eddy currents. An additional 0.5 ms delay between the EPI read gradient dephasing pulse and first echo further reduced Nyquist ghost artifact when using zero moment phase correction instead of using dual-polarity GRAPPA image reconstruction^82^.

#### BOLD 2D EPI.

Whole-brain EPI was collected using a 2D SMS sequence GRAPPA 4, multiband 3, FOV/2 controlled aliasing^83^, TE of 22 ms, TR of 7,500 ms, ES of 0.7 ms, readout bandwidth of 1,562 Hz, FOV 192 mm, matrix size 320 × 320, anterior–posterior phase encoding direction. Images were collected with oblique slices roughly aligned to the anterior commissure-posterior commissure line. Comparison data were collected on a Siemens MAGNETOM 7 T Plus fitted with an SC72 gradient coil (absolute Gmax 70 mT m^−1^, nominal Gmax 40 mT m^−1^, SR 200 T m^−1^ s^−1^) and 32-ch receive coil (Nova Medical), with matched parameters except for ES of 1.21 ms, readout bandwidth of 920 Hz and TE 35 ms.

#### BOLD 3D EPI.

A segmented 3D EPI sequence using random *k*-space sampling for greater undersampling efficiency in acceleration^35,36^ was used for whole-brain BOLD imaging, shown in Fig. 4d. The sequence consisted of 192 partitions across the *kz* axis, TE of 21 ms, TR of 5 s, in-plane FOV 180 × 144 mm, in-plane matrix 320 × 256, with 0.56 mm isotropic resolution (using a total of 12-fold acceleration of 3 (in-plane) × 4 (through-plane) and multishot 2 segmentation on the in-plane phase encode (anterior–posterior direction) axis combined with PF 0.75). This segmented 3D sequence sampling was originally created for 3D GRASE fMRI^36^ and was extended to 3D-EPI^35^. To take advantage of the flexibility in spatio-temporal space, the images were unfolded using a temporally regularized reconstruction. BOLD activations were assessed using a visuomotor task (flashing checkerboard and finger-tapping).

A segmented 3D EPI sequence variant that combines blipped-controlled aliasing with multishot segmentation^34^ was used for ultra-high-resolution BOLD imaging shown in Fig. 4e. The sequence protocol that was used for functional data acquisitions that consisted of 42 partitions was: TE 18 ms, PF 0.75, in-plane FOV 90 × 180 mm, in-plane matrix 256 × 512, slice thickness 0.35 mm and in-plane resolution 0.35 × 0.35 mm and right–left phase encoding direction. One lot of threefold undersampling with a controlled aliasing in parallel imaging results in higher acceleration (CAIPIRINHA, CAIPI for short) shift of one in partition direction combined with multishot six-segmentation resulted in a CAIPI trajectory with large phase encode blips (six) and no partition blips^34^. These scan protocols were initially developed for high-resolution VASO imaging of V1, and to achieve 0.35 mm resolution the phase-encoded FOV (left–right) was restricted to 50% of the readout FOV (head to feet) and slices were acquired coronally across the occipital pole as has been applied previously^32^. To maintain SNR for functional imaging at such high resolutions, NORDIC denoising was applied^59^. BOLD activations were assessed using a flashing checkerboard task.

#### Multiecho BOLD EPI.

Data were collected using the MultiBand (SMS) EPI 2D BOLD sequence, distributed via a Consumer-to-Producer agreement from the Center for Magnetic Resonance Research, University of Minnesota, ported onto the MAGNETOM Terra Impulse edition NexGen 7 T scanner (VE12U-AP02). EPI images were acquired at 1.6 mm resolution using a range of different ESs, leading to a range of different TEs. Common parameters across scans were: TR 2 s, SMS 3, GRAPPA 3, PF 0.75, 84 slices and anterior–posterior phase encoding direction. New images are acquired at two resolutions, one matching the 1.6 mm resolution with weaker gradient coil performance (80 mT m^−1^, 200 T m^−1^ s^−1^) and at 1.16 mm resolution using the Impulse gradient coil performance, achieving 2.5 times greater volumetric resolution. Each image at later TEs has a characteristic signal decay depending on the underlying tissue T2*, which can be affected by susceptibility in areas such as air–bone interfaces. Images collected at different TEs can be optimally combined to maximize tSNR and reduce signal dropout across different brain regions^40,42^.

#### CBV-based fMRI.

VASO EPI 0.64. Whole-brain VASO data were acquired using a segmented IR 3D EPI sequence^33,34^ using a 4 × 2 shot-selective CAIPI trajectory^84^ with a phase encode CAIPI shift of two. Parameters were as follows: 0.64 mm isotropic resolution, volume TR 4.2 s, TE 16 ms, ES 0.69 ms, readout bandwidth of 1,592 Hz, FOV 200 × 200 mm, matrix size 314 × 314, slices 180 and posterior–anterior phase encoding direction. The phase correction approach of dual-polarity EPI was used by alternating the polarity of the EPI switched read gradient waveform on alternate TRs^85^. To fulfill the VASO blood nulling condition despite T1 relaxation along the 3D EPI readout, four inversion pulses were used for each pair of BOLD and VASO *k*-space volume. Activations were assessed using a video-watching task.

#### VASO EPI 0.45/0.39.

The same sequence^33,34^ was used for VASO in a thin slab. The protocol for functional data acquisitions consisted of 18 partitions, one lot of threefold undersampling with a partition CAIPI shift of one and multishot six-segmentation (CAIPI trajectory without partition blips). Further parameters for 0.39 and 0.45 mm isotropic resolution were: TE 19 and 23 ms, PF 0.75 with projection onto convex set reconstruction with eight iterations, square in-plane matrix 374 × 462 and a right–left phase encoding direction. Activations were assessed using a flashing checkerboard task.

#### Diffusion imaging.

Participants were scanned using the 96-ch Rx coil. The diffusion EPI sequence used a fast low-angle excitation echo-planar technique^86^ reference scan for improved robustness to motion^46^. In total, five diffusion scans (6–10 min each) were acquired from a single participant using the following parameters: 53 slices, 120 mm FOV, GRAPPA 3, PF 0.75, 73 diffusion directions (including 8*b* = 0, 32*b = b*_max_/2, and 33*b = b*_max_). Two of the scans had their diffusion encoding gradients run at maximum gradient coil performance while another two were run with parameters relaxed to match the X-ray gradient coil (that is, Siemens Terra) (80 mT m^−1^, 200 T m^−1^ s^−1^) performance (with either *b*_max_ of 3,000 or 10,000 s per mm^2^ at 1.25 mm isotropic resolution). Of these four scans, the EPI readout trains were held constant at X-ray gradient coil performance to allow for direct SNR comparison of the faster diffusion encoding times. The fifth diffusion scan was acquired at 0.8 mm isotropic resolution with a *b*_max_ of 1,000 s mm^−1^, TE of 70 ms and TR of 6,000 ms. Data were processed using FSL 5.0.11 including the eddy, dtifit and bedpostx tools^87^.

Based on the Stejskel–Tanner equation *b = G*^2^ × *t*^3^ relationship, an improvement in diffusion encoding amplitude *G* by a factor of 2.5 (that is, 80 to 200 mT m^−1^) will reduce the diffusion encoding time (*t*), and thus TE, by a factor of 0.54. This factor of reduction in TE was observed at *b* = 10,000 with the Impulse gradient coil, indicating that the full 200 mT m^−1^ was used at high duty cycles. Smaller reductions in TE are expected at lower *b* values since more time is spent on gradient ramps than the flat top. Shortening the EPI echo train would allow for an additional roughly 5 ms reduction in TE. While this would significantly reduce geometric distortions, the increase in readout bandwidth would counter the SNR benefits in this case and so was not done here.

#### Structural imaging.

##### QSM.

Acquisition parameters for the multiecho GRE fast low-angle shot were: FA = 15, B0 = 6.9809, TR = 35 ms, TE = 8.25, 15.23, 23.46 ms, acceleration in-plane (right–left phase encode) five times through plane 2, matrix size 1,024 × 1,022 × 119, nominal resolution 0.21 × 0.21 × 1.5 mm^3^. Offline GRAPPA was performed to combine complex data from each coil. The phase of each channel was unwrapped using a Laplacian-based method. Variable-kernel sophisticated harmonic artifact reduction for phase data approach was used to remove background field for each echo. QSM was calculated using STreaking Artifact Reduction for QSM (STAR_QSM) method using averaged tissue phase from all three echoes using the software STI Suite (UC Berkeley).

#### MR fingerprinting.

Whole-brain (FOV 220 × 220 × 220 mm^3^) 0.56 mm T1 and T2 maps were obtained at 560 μm isotropic resolution using 3D MR fingerprinting with tiny-golden-angle-shuffling spiral-projection trajectory^48^, using 200 T m^−1^ s^−1^ for a scan time of 4 min. Additional B0 and B1+ maps were obtained using product sequences with matched FOV at 4-mm resolution.

#### GRE scout imaging.

To show spatial coverage of the Impulse gradient coil, images were collected in three orthogonal planes through midline structures of the brain and neck. TR and TE = 10 and 3 ms, FOV of 250 × 250 mm^2^, matrix size of 900 × 1,000, slice thickness of 1.5 mm, three slices, readout bandwidth of 325 Hz per pixel, flip angle of 10° (directions of phase encode were axial/anterior–posterior, sagittal/anterior–posterior, coronal/right–left), TA = 41 s.

#### MRA.

Time-of-flight MRA images were acquired with 0.4 mm isotropic resolution, TR and TE = 13 and 5.47 ms, FOV = 200 × 158.1 × 98.4 mm^3^, matrix size of 392 × 496, four slabs, 72 slices per slab, FA = 17, phase and slice partial Fourier of 7 and 8, GRAPPA 3, 70% tilted optimized nonsaturating excitation ramp^88^, readout bandwidth of 120 Hz per pixel, TA = 10 min and 19 s.

## Extended Data

## Figures and Tables

**Table 1 ∣:** Technical specifications of the Impulse gradient coil

Parameter	Value		
Coil geometry	Three-step front design, from 600 to 440 mm inner diameter (weight 1,060 kg)		
Performance	Gmax of 200 mT m^−1^; SR of 900 T m^−1^ s^−1^ (1,100 T m^−1^ s^−1^ maximum)		
Gradient power amplifier	1,200 A, 2,250 V		
Peak acoustic noise (NEMA MS 4 MGAN) (dBa)	120.6 (all axes combined)		
Cooling	31.7°C at high duty cycle, 80 mT m^−1^ for 30 min		
Active E-shims	First- and second-order harmonics		
	*x*	*y*	*z*
Linearity in 20-cm-diameter sphere volume (%)	5.73	6.32	5.64
Sensitivity (mT m^−1^ A^−1^)	0.16	0.16	0.16
Maximum positional error (% of FOV)	5.7	6.3	5.6
Maximum pixel size error (% of nominal pixel)	17.8	18.7	24.3
Inductance (μH)	315	383	315
a.c. resistance (mΩ at 1 kHz)	55	59	48
Losses at 500 *A* (kW)	13.7	14.7	12
Field at cryostat at 1,200 A (root mean square) (mT)	0.4	0.7	0.4
Net force at 7 T at 1,200 A (N)	143	155	8
Net torque at 7 T at 1,200 A (Nm)	28	150	0

## Data Availability

Sub-0.1 ml VASO fMRI data are available at OpenNeuro (https://doi.org/10.18112/openneuro.ds003850.v2.0.0). Functional, diffusion and susceptibility weighted imaging data are available at OpenNeuro (https://doi.org/10.18112/openneuro.ds004710.v1.0.0). All other data are available from the corresponding author upon reasonable request.
