# Supplementary material for: Next-generation MRI scanner designed for ultra-high-resolution human brain imaging at 7 Tesla
Source: Nat Methods. 2023 Nov 27;20(12):2048–57. doi: 10.1038/s41592-023-02068-7 (PMC10703687; doi:10.1038/s41592-023-02068-7)
Supplement: Supplementary file 1 [file 41592_2023_2068_MOESM1_ESM.pdf]

nature portfolio

Corresponding author(s): David Feinberg

Last updated by author(s): Aug 21, 2023

Reporting Summary

Nature Portfolio wishes to improve the reproducibility of the work that we publish. This form provides structure for consistency and transparency in reporting. For further information on Nature Portfolio policies, see our Editorial Policies and the Editorial Policy Checklist.

Statistics

For all statistical analyses, confirm that the following items are present in the figure legend, table legend, main text, or Methods section.

n/a	Confirmed	
☐	☒	The exact sample size (*n*) for each experimental group/condition, given as a discrete number and unit of measurement
☐	☐	A statement on whether measurements were taken from distinct samples or whether the same sample was measured repeatedly
☐	☒	The statistical test(s) used AND whether they are one- or two-sided<br>*Only common tests should be described solely by name; describe more complex techniques in the Methods section.*
☒	☐	A description of all covariates tested
☒	☐	A description of any assumptions or corrections, such as tests of normality and adjustment for multiple comparisons
☐	☒	A full description of the statistical parameters including central tendency (e.g. means) or other basic estimates (e.g. regression coefficient) AND variation (e.g. standard deviation) or associated estimates of uncertainty (e.g. confidence intervals)
☐	☒	For null hypothesis testing, the test statistic (e.g. *F*, *t*, *r*) with confidence intervals, effect sizes, degrees of freedom and *P* value noted<br>*Give P values as exact values whenever suitable.*
☒	☐	For Bayesian analysis, information on the choice of priors and Markov chain Monte Carlo settings
☒	☐	For hierarchical and complex designs, identification of the appropriate level for tests and full reporting of outcomes
☒	☐	Estimates of effect sizes (e.g. Cohen's *d*, Pearson's *r*), indicating how they were calculated

Our web collection on statistics for biologists contains articles on many of the points above.

Software and code

Policy information about availability of computer code

Data collection	Data were collected using Siemens scanners operating with software version VE12U. PsychoPy v2022.2.5 was used for stimulus presentation.
Data analysis	The following software were used for data analysis FSL v.5.0.1, mrTools v4.7, ANTs v2.1.0, LAYNII v2.2.1, AFNI v.22.0.06, SPM v12, STI SUITE v1.1, NORDIC v1.1

For manuscripts utilizing custom algorithms or software that are central to the research but not yet described in published literature, software must be made available to editors and reviewers. We strongly encourage code deposition in a community repository (e.g. GitHub). See the Nature Portfolio guidelines for submitting code & software for further information.

Data

Policy information about availability of data

All manuscripts must include a data availability statement. This statement should provide the following information, where applicable:

- Accession codes, unique identifiers, or web links for publicly available datasets
- A description of any restrictions on data availability
- For clinical datasets or third party data, please ensure that the statement adheres to our policy

Sub 0.1ml VASO fMRI data are available at OpenNeuro (https://doi.org/10.18112/openneuro.ds003850.v2.0.0). Functional, diffusion and susceptibility weighted

imaging available at OpenNeuro (https://doi.org/10.18112/openneuro.ds004710.v1.0.0). All other data are available from the corresponding author upon reasonable request.

Human research participants

Policy information about studies involving human research participants and Sex and Gender in Research.

Reporting on sex and gender	Sex and gender were not considered as part of the study, as hardware improvements would apply to all participants regardless of sex and gender.
Population characteristics	A total of 9 subjects (3 female, mean age 32.22 years, mean height 171.6cm, mean weight 64.46kg) were scanned
Recruitment	Subjects were recruited from the local population around UC Berkeley. This may have biased the participants towards higher education demographics but would have no effect on the results.
Ethics oversight	The local IRBs at UC Berkeley, San Francisco Veterans Administration and the Martinos Center for Biomedical Imaging approved the protocols used.

Note that full information on the approval of the study protocol must also be provided in the manuscript.

Field-specific reporting

Please select the one below that is the best fit for your research. If you are not sure, read the appropriate sections before making your selection.

☒ Life sciences ☐ Behavioural & social sciences ☐ Ecological, evolutionary & environmental sciences

For a reference copy of the document with all sections, see nature.com/documents/nr-reporting-summary-flat.pdf

Life sciences study design

All studies must disclose on these points even when the disclosure is negative.

Sample size	Power analyses were not performed as this is a demonstration representative data on the NexGen 7T system and not a test of specific hypotheses. Sufficient subjects were recruited to complete the scans required to demonstrate the benefits of the NexGen 7T across a number of different scan types.
Data exclusions	No data were excluded.
Replication	Figure 4b shows the replicability of the findings on the NexGen 7T across multiple sessions. Supplemental Figure 1 shows the consistency of the SNR improvements using the high-channel arrays across multiple subjects.
Randomization	Randomization not applicable since there were no groups in this study
Blinding	Blinding not applicable since there were no groups in this study.

Reporting for specific materials, systems and methods

We require information from authors about some types of materials, experimental systems and methods used in many studies. Here, indicate whether each material, system or method listed is relevant to your study. If you are not sure if a list item applies to your research, read the appropriate section before selecting a response.

Materials & experimental systems

n/a	Involved in the study	
☒	☐	Antibodies
☒	☐	Eukaryotic cell lines
☒	☐	Palaeontology and archaeology
☒	☐	Animals and other organisms
☒	☐	Clinical data
☒	☐	Dual use research of concern

Methods

n/a	Involved in the study	
☒	☐	ChIP-seq
☒	☐	Flow cytometry
☐	☒	MRI-based neuroimaging

Magnetic resonance imaging

Experimental design

Design type	Task (block design) and resting state
Design specifications	BOLD 3D EPI: Visuomotor task (8hz Flashing checkerboard and finger tapping), 30s blocks (15s on/tapping, 15s off/rest), 16 blocks, single run<br><br>Ultra-high resolution BOLD 3D EPI: Flashing checkerboard task (8hz), 30s blocks (15s on, 15s off), 20 blocks, two runs.<br><br>Whole brain VASO: Movie watching task. Subject watched multiple runs of a 10min movie clip from the Human Connectome Project. 5 scans in session 1, 4 scans in session 2.<br><br>Ultra-high resolution VASO: Flashing checkerboard task (8hz), 1 min blocks (30s on, 30s off), 3 15-minute runs per session.
Behavioral performance measures	Fixation only tasks, compliance measured by self-reporting

Acquisition

Imaging type(s)	Functional, Structrual, Diffusion
Field strength	7 Tesla
Sequence & imaging parameters	SNR and g-factor comparisons<br><br>Receive SNR measurements used a whole-brain 2D proton-density weighted gradient-echo sequence with a nominal flip-angle of 90° to limit the impact of B1+ inhomogeneities on the signal intensity [TR/TE/flip angle (FA) = 5 s/3.82 ms/90°, slice = 2 mm, matrix = 256x88, FOV = 256x176 mm2, readout bandwidth (BW) = 335 Hz/pixel, TA = 7:22 min]. Noise covariance information was acquired using the same pulse sequence, but without RF excitation. The excitation flip angle (FA) maps were acquired using a pre-conditioning saturation pulse with a turbo-flash readout [TR/TE/FA = 5 s /2.02 ms /90°, slice = 1.5 mm, matrix = 256x88, FOV = 256x128 mm2, BW = 335 Hz/pixel, Turbo factor = 128].<br>BOLD imaging<br><br>BOLD 2D EPI: Whole brain EPI was collected using a WIP 2D SMS sequence GRAPPA 4, SMS 3, FOV/2 controlled aliasing, Dual-Polarity GRAPPA image reconstruction, TE 22 ms, TR 7500 ms, ES 0.7 ms, BW 1562Hz, FOV 192mm, matrix size 320x320. Comparison data was collected on a Siemens Magnetom 7T Plus fitted with an SC72 gradient coil (70mT/m, 200T/m/s) and 32ch receive coil (Nova Medical), with matched parameters except: ES 1.21 ms, BW 920hz, TE 35 ms.<br><br>BOLD 3D EPI: A segmented 3D-EPI sequence utilizing random k-space sampling for greater undersampling efficiency in acceleration was used for whole-brain BOLD imaging. 192 partitions across the kz axis, in-plane FOV 180 mm x 144 mm, in-plane matrix 320x256, slice thickness 0.56 mm and in-plane resolution 0.56 mm x 0.56 mm using 12-fold acceleration of 3 (in-plane) x 4 (through-plane) and multi-shot 2 segmentation on the in-plane axis combined with PF=6/8.Images were unfolded using a temporally regularized reconstruction.<br><br>Ultra-high resolution 3D-EPI: Blipped-controlled aliasing with multi-shot segmentation, 42 partitions, TE 18 ms, PF 6/8, in-plane FOV 90 mm x 180 mm, in-plane matrix 256x512 , slice thickness 0.35mm, and in-plane resolution 0.35 mm x 0.35 mm. 1x3-fold undersampling with a CAIPI shift of 1 in partition direction combined with multi-shot 6 segmentation resulted in a CAIPI trajectory with large phase encode blips (6) and no partition blips. To achieve 0.35 mm resolution the phase encoded FOV (left-right) was restricted to 50% of the readout FOV (head-feet) and slices were acquired coronally across the occipital pole as has been applied previously.<br><br>Multi-Echo BOLD EPI: Data were collected using the Multi-Band (SMS) EPI 2D BOLD sequence, distributed via C2P from CMRR ported onto the MAGNETOM Terra Impulse edition NexGen 7T scanner (VE12U-AP02). EPI images were acquired at 1.6 mm resolution using a range of different echo spacings, leading to a range of different TEs. Common parameters across scans: TR = 2s, SMS 3, GRAPPA 3, PF 6/8, 84 slices.<br><br>CBV Imaging<br><br>VASO EPI 0.64: Whole-brain VASO data were acquired using a segmented IR 3D-EPI sequence using a 4x2 shot-selective CAIPI trajectory with a phase encode CAIPI shift of 2. Parameters: 0.64 mm isotropic resolution, Volume TR = 4.2s, TE=16 ms, echo spacing=0.69 ms, BW=1592 Hz, FOV= 200 mm x 200 mm, matrix size 314 x 314, 180 slices. The phase correction approach of Dual-polarity EPI was employed by alternating the polarity of the EPI switched read gradient waveform on alternate TRs. In order to fulfill the VASO blood nulling condition despite T1-relaxation along the 3D EPI readout, four inversion pulses were used for each pair of BOLD and VASO k-space volume.<br>A complete list of scan parameters are avaliable on: https://github.com/layerfMRI/Sequence_Github/blob/master/Terra_protocolls/Berkeley_NextGen/0.6mm_protocol_WB.pdf<br><br>VASO EPI 0.45/0.39: The same sequence was used for VASO in a thin slab. The protocol for functional data acquisitions consisted of 18 partitions, 1x3-fold undersampling with a partition CAIPI shift of 1 and multishot 6 segmentation (CAIPI trajectory without partition blips). Further parameters for 0.39/0.45mm isotropic resolution were: TE 19 ms/23 ms, PF 6/8 with POCS reconstruction with 8 iterations, square in-plane matrix 374/462.

March 2021

A complete set of scanning parameters are avaliable on https://github.com/layerfMRI/Sequence_Github/blob/master/Terra_protocolls/Berkeley_NextGen/Renzo.pdf

Diffusion Imaging

The CMRR C2P Diffusion EPI sequence with a FLEET reference scan was used for improved robustness to motion. In total, five diffusion scans (6-10 mins each) were acquired from a single subject using the following parameters: 53 slices, 120 mm FOV, GRAPPA 3, PF 6/8, 73 diffusion directions (including 8 b0s, 32 b=bmax/2, and 33 b=bmax). Two of the scans were run at maximum gradient performance while another two were run with parameters relaxed to match XR gradient (i.e. Siemens Terra) (80mT/m, 200T/m/s) performance (with either bmax = 3,000 or 10,000 s/mm2 at 1.25 mm isotropic resolution. A diffusion scan was acquired at 0.8 mm isotropic with a bmax of 1000 s/mm , TE=70 ms and TR=6000 ms. Data were processed using FSL 5.0.11 and the eddy, dtifit, and bedpostx tools94.

Structural Imaging

QSM: Acquisition parameters for the multi-echo GRE FLASH were: FA = 15, B0 = 6.9809, TR = 35 ms, TE = 8.25, 15.23, 23.46 ms, IPAT = 5 x 2, Matrix size = 1024x1022x119, Resolution = 0.21x0.21x1.5 mm3 Offline GRAPPA was performed to combine complex data from each coil.

MRF: MR fingerprint imaging (MRF) Whole-brain (FOV 220x220x220mm3) 0.56 mm T1 and T2 maps were obtained at 560 um isotropic resolution using 3D MRF with tiny-golden-angle-shuffling spiral-projection (TGAS-SPI) trajectory50, using 200 T/m/s for a scan time of 4min. Additional B0 and B1+ maps were obtained using product sequences with matched FOV at 4-mm resolution.

Area of acquisition: Whole brain and occipital cortex

Diffusion MRI: ☒ Used ☐ Not used

Parameters: 73 diffusion directions (including 8 b0s, 32 b=bmax/2, and 33 b=bmax). No cardiac gating.

Preprocessing

Preprocessing software: Data were preprocessed using AFNI v.22.0.06, SPM v12, ANTs v2.1.0 and LAYNII v2.2.1

Normalization: Data were not normalized, analyses performed in functional space to avoid issues of blurring and interpolation.

Normalization template: Data were not normalized

Noise and artifact removal: Data were motion corrected, but noise and artifact signals were not regressed. Ultra-high resolution 3D EPI was denoised using NORDIC.

Volume censoring: Data were not censored

Statistical modeling & inference

Model type and settings: Mass univariate

Effect(s) tested: Visual activation in a flashing checkerboard task

Specify type of analysis: ☐ Whole brain ☐ ROI-based ☒ Both

Anatomical location(s): Occipital Cortex

Statistic type for inference (See Eklund et al. 2016): Voxelwise

Correction: No correction was used

Models & analysis

n/a	Involved in the study
☐	☒ Functional and/or effective connectivity
☒	☐ Graph analysis
☒	☐ Multivariate modeling or predictive analysis

Functional and/or effective connectivity: Whole brain Vaso analysis: Time series were extracted for the common ICA networks, orthogonalised and used as regressors for activation maps.
sub-microliter VASO analysis: AFNI GLM, with 0.2mm layer-smoothing (LayNii) and 3dClust (AFNI) at z-score 1.5 with 200 voxels.
